# Predictors and consequences of diet composition in a declining generalist aerial insectivore

**DOI:** 10.1007/s00442-026-05901-w

**Published:** 2026-06-04

**Authors:** Jennifer J. Uehling, Conor C. Taff, Jennifer L. Houtz, Paige M. Becker, Allison S. Injaian, Maren N. Vitousek

**Affiliations:** 1 Department of Ecology and Evolutionary Biology, Cornell University, Ithaca, USA; 2 Cornell Lab of Ornithology, Ithaca, USA; 3 Department of Biology, West Chester University, West Chester, USA; 4 Department of Biology, Allegheny College, Meadville, USA; 5 Department of Biology, Boston University, Boston, USA; 6 Odum School of Ecology, University of Georgia, Athens, USA

**Keywords:** Birds, Foraging ecology, DNA metabarcoding, Insects, Diet quality

## Abstract

For generalist animals that have a broad range of food choices, the specific diet characteristics that are important for health and fitness are often unclear. Here, we examine whether individual variation in diet composition predicts nestling mass and fledge success, and we identify potential drivers of variation in diet composition, in a declining generalist aerial insectivore, the tree swallow (*Tachycineta bicolor*). We collected morphological measurements, feces, and fledge success information from adult and nestling swallows. We then used DNA metabarcoding of 429 fecal samples to characterize swallow diets. We focused on two diet characteristics that may be important for generalists: dietary diversity and prevalence of nutritionally important diet items, specifically aquatic insects, which are essential to tree swallows. We predicted that nestlings with more diverse diets that were composed of a higher proportion of aquatic arthropods would be more likely to survive to fledging, and that adults’ phenotypes and their stage in the breeding season would be associated with both their own diets and the diets of their nestlings. Swallow nestling dietary diversity strongly predicted fledge success, but we found no link between dietary aquatic arthropod content and nestling fledging. Adult phenotype did not predict nestling diet, but during the provisioning period, adult females with lower body mass had more diverse diets. Because this study occurred during a single breeding season in one geographic area, future work should further explore the patterns observed here, and especially examine the importance of dietary diversity for wild generalist species across years and localities.

**Supplementary Information:**

The online version contains supplementary material available at 10.1007/s00442-026-05901-w.

## Introduction

Inter-individual variation in diet can have large consequences for individual fitness and population health. Differences among diets can affect survival (Cruz-Rivera and Hay 2000; Cook et al. 2004; Harrison et al. 2014; Belgrad and Griffen 2016; Kutzer et al. 2018) and reproductive performance (Kumar and Ramakrishna Rao 1999; Cruz-Rivera and Hay 2000; Cook et al. 2004; Griffen 2014; Harrison et al. 2014; Belgrad and Griffen 2016; Kutzer et al. 2018) across species. Adults’ foraging decisions also affect the health and fitness of their young via offspring provisioning (Resano-Mayor et al. 2014; Guillod et al. 2016; Christensen-Dalsgaard et al. 2018).

Given the importance of diet, the choices animals make during foraging have fascinated scientists for decades. Numerous theories about diet selection and foraging behavior have been proposed and tested empirically. For example, optimal foraging theory (Emlen 1966; MacArthur and Pianka 1966) can predict animals’ decisions about what to eat and where to forage under different contexts. Similarly, the field of nutritional ecology, which has developed in parallel with optimal foraging theory, seeks to identify and understand the effects of “foraging currencies” such as nutrients and plant secondary metabolites, and how they affect consumer performance and fitness (Raubenheimer et al. 2009; Raubenheimer and Simpson 2018).

Despite the large body of literature around what animals eat and why, the fitness consequences of individual variation in diet are often poorly understood. This is particularly true for wild generalists; unlike specialists, whose diet choices are constrained, generalists can choose from a variety of dietary options and are less likely to experience scarcity of suitable food (Schleuter and Eckmann 2008; Terraube et al. 2011). However, not all palatable food items are of equivalent value. Within populations, individual generalists often differ remarkably in their diet composition (Bolnick et al. 2003, 2007; Woo et al. 2008; Smith et al. 2011), yet it can be challenging to determine whether certain items or diet characteristics are more important for health and fitness than others. Most studies on the fitness consequences of diet variation in generalists have been done in captive populations with highly limited food items available (Kumar and Ramakrishna Rao 1999; Cruz-Rivera and Hay 2000; Harrison et al. 2014; Belgrad and Griffen 2016; Kutzer et al. 2018). Thus, it is unclear how their findings might translate to wild settings, where there is a myriad of food options available.

Both dietary diversity (DeMott 1998; Lefcheck et al. 2013; Resano-Mayor et al. 2014) and nutrient content (Karasov and Martinez 2007; Twining et al. 2016a, 2018a) may be particularly important for generalists’ health and fitness and could play a role in an organism’s foraging choices. Dietary diversity may be beneficial because different foods complement each other nutritionally (DeMott 1998). Additionally, dietary diversity can impact host physiological traits such as microbiome diversity (Bolnick et al. 2014; Heiman and Greenway 2016; Tiede et al. 2017; but see Kartzinel et al. 2019; Wang et al. 2021), which can be important for overall health and fitness (Turnbaugh and Gordon 2009; Bolnick et al. 2014). Furthermore, diverse diets may be more likely to contain currently unrecognized but vital prey items with important nutrients. A meta-analysis, primarily of lab experiments, found some evidence that mixed diets lead to better growth and reproduction (Lefcheck et al. 2013). However, results in wild birds are inconsistent, with studies finding positive relationships (Whitfield et al. 2009; Margalida et al. 2012), negative relationships (Resano-Mayor et al. 2014; Lourenço et al. 2015), or no relationship (Serrano-Davies and Sanz 2017) between dietary diversity and reproductive success. The presence of specific nutrients in the diet may also affect health and fitness. For example, some essential macronutrients, such as certain fatty acids, may be relatively rare in the environment and cannot be synthesized de novo (Karasov and Martinez 2007; Twining et al. 2016a).

Here, we examine the diet of a generalist bird that breeds across much of North America (Winkler et al. 2020), the tree swallow (*Tachycineta bicolor*), to understand whether diet composition (dietary diversity and dietary nutrient content) is associated with nestling body mass and fledging success, and to identify potential drivers of individual variation in diet composition. Tree swallows are aerial insectivores, meaning they eat flying insects (Winkler et al. 2020). Though tree swallows may occasionally eat other prey items (such as mollusks and berries), these prey items are consumed less commonly (Winkler et al. 2020), and so we focus on flying insects here. Aerially insectivorous birds are broadly declining across North America (Rosenberg et al. 2019), especially in the Northeast (Nebel et al. 2010; Smith et al. 2015) where this study was conducted. One possible reason behind this decline is reductions in flying insect populations (Nebel et al. 2010; Spiller and Dettmers 2019). A more thorough understanding of the diets of tree swallows, and the relationships between dietary variation and fitness, may help to illuminate whether changing insect availability plays a role in the decline of aerial insectivores (Bellavance et al. 2018).

As generalists, tree swallows may depend on both dietary diversity and dietary nutrient content for health and breeding success. However, we still lack an understanding of whether variation in dietary diversity and nutrient content at the individual level predicts variation in fitness outcomes in wild swallows. Previous work has demonstrated that tree swallows rely on a broad variety of prey items (McCarty and Winkler 1999; Beck et al. 2013; Bumelis et al. 2022), suggesting that diverse diets may be valuable to these birds. However, we are not aware of studies that have tested whether the diversity of prey ingested affects proxies of fitness in tree swallows or other aerial insectivores at the individual level. Additionally, highly unsaturated omega-3 fatty acids (HUFAs), macronutrients involved in cardiovascular and immune function, neuronal development, and cognitive and visual function (Jump 2002; Brenna and Carlson 2014), have been identified as an important and limiting macronutrient in the diets of tree swallows (Twining et al. 2016b, 2018a, b). In the wild, flying insects that have their immature stage in water (henceforth aquatic insects) have much higher levels of HUFAs (Hixson et al. 2015; Twining et al. 2018a, 2019), and in years with higher abundances of aquatically-originating aerial insects, tree swallows have higher reproductive success (Twining et al. 2018b). Elgin (2019) suggests that a greater ratio of aquatic insects to terrestrial insects in individual nestling tree swallows’ diets positively predicts their mass; however, beyond this work, no studies have yet tested whether HUFA diet content predicts the performance and fitness of individual tree swallows in the wild.

In this study, we explore whether individual dietary variation in nestling tree swallows is associated with mass and fledge success, and potential causes of individual dietary variation in adult and nestling tree swallows. We characterized swallow dietary variation using DNA metabarcoding targeting the mitochondrial cytochrome C oxidase subunit I (COI) gene of arthropod prey items found in fecal samples. Thus, though insects are the primary diet items consumed by tree swallows, we focused on the broader classification of arthropod diet items in this work. Specfically, we tested the following:

1. **Diet**,** nestling mass**,** and fledging success**: We tested whether dietary diversity and dietary aquatic arthropod content (which is associated with dietary HUFA content) play a role in nestling tree swallow health and fledge success. We predicted that nestlings with more diverse diets and greater proportions of their diets composed of aquatic arthropods would show greater mass (e.g., Elgin 2019) and a higher probability of fledging.
2. **Predictors of diet composition in nestlings and adults**: We tested whether the phenotypes of foraging adults predict their own diets – as has been found for other species (Magalhães de Oliveira et al. 2020; Hall et al. 2021; Shaner et al. 2021) – and the diets of their offspring. We also tested whether study site and nestling age predicted diets.
  - **Adult phenotype**: Because longer wings may be associated with longer-distance flight (Marchetti et al. 1995; Milá et al. 2008), we predicted that females with longer wings would perform more extended foraging bouts over many types of habitats, and therefore consume, and feed their nestlings, more diverse diets. Additionally, we predicted that female mass would be associated with diet composition. Females exhibit adaptive mass loss from the incubation period through the peak provisioning period, which is thought to facilitate more efficient foraging (Boyle et al. 2012). Therefore, we predicted that, during provisioning, adult female mass would be negatively associated with dietary diversity and the proportion of the diet composed of aquatic arthropods in both adult females and their nestlings.
  - **Study site**: We tested whether study site predicted diet composition in adult and nestling tree swallows. We predicted that there would be no relationship between study site and dietary diversity because we were unaware of specific ecological factors that would drive arthropod diversity differences between study sites. However, some of the sites have nest boxes directly adjacent to water, and so we predicted that the diets of adults and nestlings at these sites would contain a higher proportion of aquatic arthropods.
  - **Nestling age**: We tested whether nestlings at days 6, 12, and 15 showed differences in their diet composition. We predicted that day 12 nestlings would have higher proportions of aquatic arthropods in their diets than day 6 and day 15 nestlings. Tree swallows experience exponential growth from about days 7–12 after hatching (Zach and Mayoh 1982; Twining et al. 2018b), so foods of high nutritional value for developing nestlings may be especially important at this stage. We predicted that nestlings’ dietary diversity would not differ across age.
3. **Diet composition in adults versus nestlings**: We explored how diets differed between adult males, adult females, and nestlings during the provisioning period. We predicted that nestlings would have a higher proportion of aquatic arthropods in their diets than adults, given the known importance of HUFA-rich diets for developing nestlings (Twining et al. 2016b, 2018b), but that there would be no differences in dietary diversity between adults and nestlings. We also predicted that adult males and females would show no differences in their diet compositions because we were unaware of any sex-specific differences in nutritional needs.

## Materials and methods

### Fieldwork

We studied tree swallows during their breeding season at a long-term study area in Tompkins County, New York, USA from April through July 2019. We sampled tree swallows (159 adults and nestlings from 63 nests) from four long-term study sites across this area: Unit 1 (42.504º N, −76.466º W), Unit 2 (42.503º N, −76.437º W), Unit 4 (42.460º N, −76.365º W), and Turkey Hill (42.441º N, −76.429º W) (Figs. S1A and S1B). The two sites furthest from each other are Unit 1 and Unit 4, at approximately 10 km apart. These sites represent a spectrum of habitat types. Unit 1 and Unit 2 make up the Cornell University Experimental Ponds facilities. Combined, these two sites contain 91 artificial ponds, and each site has a large reservoir. At these two sites, nest boxes are located adjacent to ponds, and at Unit 1, some nest boxes are located on poles in the large reservoir. Unit 4 is in the middle of an agricultural area with nest boxes situated along two gravel roads that intersect agricultural fields. The area immediately around Unit 4 does not have any large, permanent bodies of water, although there are small farm ponds in the vicinity and a small intermittent stream at the site. Finally, Turkey Hill is a wet meadow located adjacent to a creek (Cascadilla Creek) that was formerly agricultural land. The nest boxes at this site are situated in successional old field, shallow emergent marsh, and shrub swamp, and are surrounded by shrubland, forest, and agricultural land (https://cornellbotanicgardens.org/location/turkey-hill-road-meadow/).

As part of the standard monitoring protocol at our long-term study site (Vitousek et al. 2018a, b), we checked each nest box every other day from late April through early July to determine clutch initiation dates, hatch dates, and nestling status. During the last few days of the nestling period, we avoided checking nest boxes so as not to initiate premature fledging. When nests reached the fledging stage (approximately 18–20 days after hatching), we checked nest boxes to determine fledge success (died vs. fledged). We also captured and sampled adult and nestling birds throughout the breeding season (April through July) on different schedules depending on the site.

All adult captures occurred between 0600 h and 1000 h. We captured adult females on day 6–7 of incubation (“mid incubation”) and 6–7 days after their nestlings hatched (“provisioning”). At Unit 1 and Unit 2, we also captured adult females on day 12 of incubation (“late incubation”). Additionally, during day 6–7 of provisioning, we aimed to catch most males at Unit 1 and Unit 2. We opportunistically caught males at Turkey Hill and Unit 4, but because we ended up catching so few males at these sites, we excluded them from all analyses. During the first adult capture, we banded each bird with a uniquely numbered USGS-issued aluminum band if they were not already banded, and we measured each bird’s flat wing length to the nearest mm. During all adult captures, we measured mass to the nearest 0.25 g using a Pesola spring scale and took blood samples from the brachial vein using venipuncture.

For nestling samples, at Unit 1 and Unit 2 we sampled nestlings on day 12 and day 15 of provisioning; at Unit 4 and Turkey Hill we sampled nestlings on day 6–7 and day 12 of provisioning. During sampling efforts on day 12 we banded each nestling with a uniquely numbered USGS-issued aluminum band and took blood samples. On days 12 and 15 we also measured the mass of each individual nestling.

During adult and nestling captures, we collected feces from each bird when possible. If the bird defecated during initial capture, we collected the fecal sample, regardless of the size. If birds did not immediately defecate upon capture, or if they produced a very small sample, for adults, we placed them in a paper bag between blood sampling events and collected any feces from the bag (adding to the initial small sample, if one was collected), and for nestlings, we pressed lightly on their abdomens to promote defecation. We took care to avoid cross-contamination between samples; for example, if multiple nestlings defecated and their fecal matter touched, we did not collect those samples. All fecal samples were placed in a 1.5 mL microcentrifuge tube, put on ice, and taken back to the field lab. Fecal samples were stored at −30º C for the remainder of the field season and then transferred to a −80º C freezer.

### Lab work

We extracted DNA from fecal samples using Qiagen DNeasy PowerSoil kits. For every 23 fecal samples extracted, we extracted one “blank” sample to serve as a kit negative control. All steps of the DNA extraction (and subsequent PCR steps) were performed on surfaces sanitized with 70% ethanol and with gloved hands sanitized with 70% ethanol. We followed the manufacturer’s protocol with a few modifications. We added phosphate buffered saline (PBS) to each sample tube to create a ratio of 0.2 g of fecal material for every 100 uL of PBS. The PBS was added to make a more liquid fecal slurry, which made the feces easier to homogenize and pipette from the sample tube to the PowerBead tube. This homogenization prevented subsampling from a specific part of the feces. For samples less than 0.2 g, we added 100 uL of PBS. After adding PBS, we homogenized the sample in the collection tube by vortexing it, and/or by using a handheld pestle homogenizer. We then transferred 100 uL of the homogenate into the PowerBead tube (100 uL of PBS for blank samples). After adding Solution C1 to each tube, we incubated the tubes at 65° C in a water bath for 30 min. We then proceeded to homogenize the samples in a bead beater, and for the remainder of the extraction, we followed the steps provided in the manufacturer’s protocol. DNA extracts were stored at −30º C until PCR.

We characterized diet by amplifying the COI gene. We selected primers BF2 (forward) and BR2 (reverse) based on performance on insect species reported in Piñol et al. (2019). More specifically, we performed a pilot test with primers from Piñol et al. (2019) that were reported to perform well with insect species, and we selected BF2 and BR2 because the PCR products produced strong bands via gel electrophoresis. We performed PCR in 10 µl triplicates (three reactions per fecal DNA extract). Each PCR reaction contained 3.75 µl Platinum Hot Start PCR Master Mix (2x), 0.1875 µl of BF2 (10x), 0.1875 µl of BR2 (10x), and 4.875 µl of nuclease-free water. We chose thermocycler conditions from Elbrecht & Leese (2017); thermocycler conditions were the following: (a) 94º C for 3:00, (b) 94º C for 30 s, (c) 50º C for 30 s, (d) 65º C for 2:30 [repeat b-d 35 times], (e) 65º C for 5:00, and (f) hold at 4º C. We checked for successful amplification (421 base pairs; Piñol et al. 2019) via gel electrophoresis on a subset of samples (1% gel at 200 V for 15 min). After PCR, we pooled the triplicate samples and stored them at −30º C until submission for sequencing. Samples were submitted to the Cornell Biotechnology Resource Center for library preparation and sequencing with Illumina MiSeq (2 × 250 bp paired end). Samples were submitted in 96-well plates. The Cornell Biotechnology Resource Center measured the concentration of DNA in nanograms per microliter for each sample, and then normalized DNA concentrations for each sample prior to sequencing.

### Diet bioinformatics pipeline

The raw sequences for each sample were processed using the AMPtk (version 1.4.0) pipeline in python (Palmer et al. 2018), closely following the workflow described on the project website (amptk.readthedocs.io/en/latest). The specific commands used in python are shown in the first section of the R script “data_filtering_script.R” (https://github.com/juehling/tres_coi_analysis_2019_ms). In brief, forward and reverse reads were merged and primer sequences were removed. We then filtered sequences based on base pair (bp) length. If sequences were less than 401 bp, we removed them from the dataset. If they were more than 441 bp, we trimmed them to 441 bp. Therefore, moving forward, our dataset only contained sequences that fell within 20 bp of our target amplicon length of 421 bp.

Data were then denoised with DADA2 (Callahan et al. 2016) by taking the processed sequences and applying a denoising algorithm that identifies amplicon sequence variants (ASVs) and models sequencing error to arrive at a list of ASVs. These ASVs were then clustered into biological operational taxonomic units (OTUs) with a threshold of 97% similarity. AMPtk selected representative sequences for each OTU as the most abundant ASV within that cluster, and this representative ASV was then used for taxonomy assignment using AMPtk’s “hybrid taxonomy algorithm” and comparing sequence data to a COI arthropod/chordate database constructed from the BOLDv4 database (https://amptk.readthedocs.io/en/latest/taxonomy.html). The hybrid taxonomy algorithm uses three alignment algorithms, and we used AMPtk’s default threshold values for each of these algorithms (Solís-García et al. 2021; Kim et al. 2022): Global Alignment to the COI arthropod/chordate database (USEARCH/VSEARCH; percent identity cutoff: 0.7), UTAX (confidence threshold: 0.8), and SINTAX (confidence threshold: 0.8). The outputs from each of these algorithms are compared, and the hybrid algorithm goes through decision points to retain results that have both high levels of taxonomy and high percent identity (see the link above for a full description of the algorithm). The taxonomy chosen by the algorithm was then applied to each ASV within the clustered OTU, thereby collapsing each OTU to a single taxonomic classification at a specific taxonomic rank.

After completing processing in AMPtk, we imported processed files into R version 4.3.1 (R Core Team, 2023), where we completed all subsequent data filtering and analyses. The processed files were merged into a single phyloseq object (McMurdie and Holmes 2013). We then filtered sequences to retain only arthropod reads. After this filtering step, we exported a list of all arthropod families present in the samples. Via internet searches and consultations with entomologists, we identified the habitat of the immature stage of each arthropod family from the following categories: aquatic, terrestrial, both, and unknown. A classification of “both” indicated that some members of the family have aquatic immature stages and some have terrestrial immature stages, and “unknown” indicated that we could not find enough information about the family to make a determination. Using the “phyloseq” package in R, we then agglomerated to family (which eliminated all reads that could not be identified at least to family) and removed all families with fewer than ten reads across all samples (Hoenig et al. 2021; Forsman et al. 2022). Next, we plotted the number of reads against the number of samples, with adult, nestling, and blank samples separated to visualize possible differences (Fig. S2). When comparing blank samples to adult and nestling samples, we found that most blank samples had fewer than 100 reads whereas most adult and nestling samples had more than 100 reads. Therefore, we chose to remove all adult and nestling samples with fewer than 100 arthropod reads per sample in an effort to remove samples with failed extractions or PCR reactions. Finally, we calculated the relative read abundance of families in each sample.

After classifying families based on their immature stage habitat and performing the filtering steps, we calculated the proportion of each sample composed of aquatic arthropods in two ways. First, we calculated proportion aquatic arthropods using the relative read abundance of aquatic arthropods in each sample. To do this, we added together all of the previously calculated relative read abundances of aquatic families in each sample. Second, we calculated the proportion aquatic arthropods using occurrence of aquatic families. For each sample, we calculated the total number of aquatic families, and then we divided this by the total number of arthropod families found in the sample.

We performed all subsequent statistical analyses with proportion aquatic arthropods calculated via relative read abundance and proportion aquatic arthropods calculated via occurrence (see Discussion for some of the strengths and weaknesses of these metrics, and a brief review of the use of relative read abundance in other diet studies). For a more thorough examination of the relationship between relative read abundance versus occurrence metrics in this dataset, please see the Supplemental Material (Online Resource 1, *Relationship between proportion aquatic arthropods via relative read abundance versus occurrence*, Fig. S3).

Finally, we used the R package vegan (Oksanen et al., 2022) to calculate Simpson’s diversity index, a metric of alpha diversity that incorporates both richness and evenness (Kim et al., 2017; Simpson, 1949), for each fecal sample.

### Statistical analyses

#### Metrics of diet composition

We performed all analyses substituting in three different variables to describe diet composition: proportion aquatic arthropods calculated via relative read abundance (PRA), proportion aquatic arthropods calculated via occurrence (PO), and alpha diversity (calculated as Simpson’s diversity index). Based on the recommendations of Warton and Hui (2011) for the analysis of proportions in ecology, we logit-transformed all proportion data using the R package “gtools” (Bolker et al. 2022) before putting them as response variables into the models.

#### General modeling practices

In all models, we used centered and standardized continuous predictor variables so that they had a mean of zero and standard deviation of one, using the “scale” function in R. This was done to make statistical outputs more intuitive and easier to interpret. We used the package “lme4” for linear mixed effects models (LMMs) and generalized linear mixed effects models (GLMMs) (Bates et al. 2015). For all LMMs, we calculated degrees of freedom using Kenward-Roger. Finally, when appropriate, we used the emmeans package (Lenth, 2022) to perform post hoc pairwise comparisons. We considered explanatory variables with p-values of less than 0.05 to be strong predictors of the response variable.

Some of the individuals sampled in this study were part of experiments in which ventral plumage color and/or perceived predation risk, or exposure to light at night, were manipulated (described in the Supplemental Material, Online Resource 1, under *Experiment details*). We included experimental treatment as a random effect in all models.

#### Diet, nestling mass, and fledging success

We used LMMs to examine the relationship between diet composition and nestling mass on nestling day 12 and day 15, with diet composition as a fixed effect and experimental treatment and nest box as random effects. We created separate models for day 12 and day 15 nestlings.

We used GLMMs to examine the relationship between diet composition and fledge success, with a binomial family and a logit link function. We removed all nestlings from the analysis that were depredated (4 nestlings). The remaining nestlings died from other causes and could be identified based on their USGS aluminum bands. We created separate models for day 12 and day 15 nestlings. For the models for day 12 nestlings, diet composition was a fixed effect and experimental treatment and nest box were random effects. For the models for day 15 nestlings, there were very few nestlings that did not fledge (died, *n* = 6; fledged, *n* = 47). Because there were so few nestlings that died after day 15, we could not estimate the random effect of nest box. Thus, for the models for day 15 nestling fledge success, diet composition was a fixed effect and experimental treatment was a random effect.

#### Predictors of diet composition for nestlings and adults

We next tested which variables predict adult and nestling tree swallow diets. We used LMMs to explore predictors of adult and nestling diets. Analyses of nestling diet included adult mass during provisioning, adult flat wing, site, brood size, and nestling age as fixed effects. Thus, these analyses excluded samples collected from nestlings whose mothers were not also captured during the provisioning period (i.e., we tried but failed to catch them). We also included experimental treatment and nest box as random effects.

Analyses of adult diet used flat wing, mass, breeding stage (mid incubation, late incubation, or provisioning), an interaction between mass and breeding stage (to account for possible changing relationships between mass and diet during different breeding stages due to adaptative mass loss), and site as fixed effects. Experimental treatment and female identity were used as random effects. For a subset of birds, to examine the possible effect of mass loss more directly, we performed an additional analysis where we examined whether mass loss from mid incubation to provisioning predicted diet composition during provisioning. For this analysis, we used females for which we had fecal samples from the provisioning capture and mass measurements from both the mid incubation and provisioning captures. Mass loss, flat wing, and site were fixed effects and experimental treatment was a random effect.

#### Diet composition in adults versus nestlings

To directly examine whether adult female, adult male, and nestling diets differed, we fit an LMM with bird category (adult female, adult male, or nestling) as a fixed effect and nest box and experimental treatment as random effects. Due to the possibility that a female’s experimental treatment might affect the behavior of her male mate, the the experimental treatment of each female was also assigned to her mate.

For this analysis we used only birds captured during the provisioning period because insect availability may change across stages of the breeding season (Twining et al. 2018b) and males were only captured during provisioning. We excluded a single male fecal sample from analyses because the bird was accidentally captured during incubation. We included nestling samples from days 6, 12, and 15.

## Results

### Sequencing results

After running the AMPtk pipeline and prior to filtering to just reads from arthropods, we successfully recovered sequences from 547 out of 548 tree swallow fecal samples. Across fecal samples, there were a total 13,404,463 reads, with a range of 15 reads to 156,209 reads in individual samples. The mean number of reads per sample was 24,505, and the median was 18,178. There were 6,599 OTUs across all fecal samples. We sequenced 26 negative control DNA extractions (“blanks”). Across blanks, there were a total of 30,174 reads, with a range of 12 reads to 9,876 reads in each individual blank. The mean number of reads per blank was 1,161, and the median was 78. There were 278 OTUs across all blanks.

After filtering to only reads from arthropods in fecal samples, we retained 4,022,012 reads, with a range of 1 read to 97,059 reads in individual samples. The mean number of reads per sample was 7,353 and the median was 1,809. We retained an average of 27.2% of the reads from each sample, with a range of 0.033% to 99.2%. There were 5,408 OTUs across all fecal samples. After filtering to only reads from arthropods in blank samples, we retained 2,658 reads, with a range of 4 reads to 1,672 reads. The mean number of reads per sample was 102, and the median was 31. There were 232 OTUs across all blanks.

After all filtering steps, including agglomerating to family and removing samples with fewer than 100 arthropod reads, our final dataset retained sequences from 429 fecal samples: 189 from adults and 240 from nestlings. Of the adult samples, 47 were from males and 142 were from females (Table S1). Of the nestling samples, 46 were from day 6 nestlings, 141 were from day 12 nestlings, and 53 were from day 15 nestlings (Table S2). Of the adults with fecal samples in the final dataset, 97 had one sample in the dataset, 28 had two samples in the dataset, and 12 had three samples in the dataset, for a total of 137 unique individuals sampled. Of the 170 banded nestlings with fecal samples in the dataset, 24 individuals had samples taken on both day 12 and day 15.

### Arthropod families present in diet

After all filtering steps, we identified 161 families in the diets of tree swallow adults and nestlings. The mean number of arthropod families per sample was 12 and the median was 11, with a range of 2 to 34 families per sample.

The families with the highest mean relative abundances across samples were Limoniidae (limoniid crane flies, mean = 0.266), Chironomidae (non-biting midges, mean = 0.170), Miridae (capsid bugs, mean = 0.047), Culicidae (mosquitoes, mean = 0.042), Tipulidae (crane flies, mean = 0.038), and Rhagionidae (snipe flies, mean = 0.036).

**Fig. 1:**
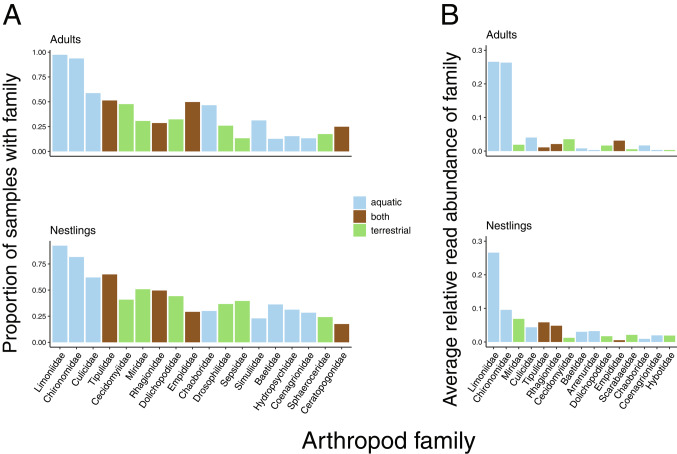
(**A**) The proportion of samples each arthropod family was detected in, with only those families detected in 0.20 or more of fecal samples shown. (**B**) The average relative abundance of arthropod families in samples, with the families with the fifteen highest average relative abundance values shown

Of these 161 families, 52 were classified as aquatic, 92 as terrestrial, 16 as both, and 1 as unknown. The unknown family, Entomobryidae, had a very low mean relative abundance across samples (0.00005). Eighteen families were present in 20% or more of samples (Fig. 1a). The four families found in the highest proportion of samples – Limoniidae, Chironomidae, Culicidae, and Tipulidae (Fig. 1a) – also had relatively high mean relative abundances across adult and nestling samples (Fig. 1b).

### Diet, nestling mass, and fledging success

For all remaining results, we indicate the proportion of the diet composed of aquatic arthropods calculated via relative read abundance with “PRA,” and the proportion of the diet composed of aquatic arthropods calculated via occurrence with “PO.”

**Fig. 2:**
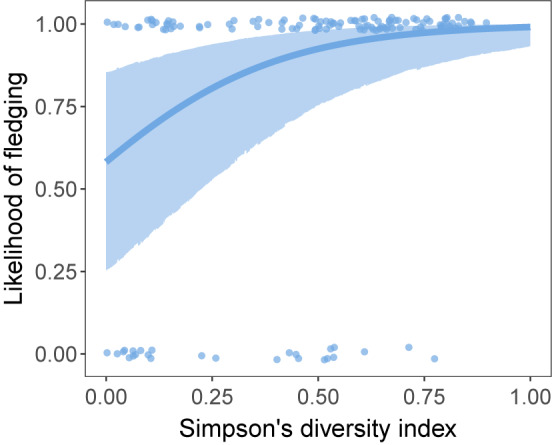
Relationship between Simpson’s diversity index of the day 12 nestling diet and the likelihood of fledging. The blue line shows the predicted relationship from the GLMM, and the shaded blue area shows the 95% confidence interval of the prediction. The blue points show raw data from individual samples

In day 12 nestlings, mass was unrelated to dietary diversity (*n* = 141, $\:\beta\:$ = 0.13, df = 108.24, *p* = 0.56) or to the proportion of the diet composed of aquatic arthropods calculated via relative read abundance (PRA: *n* = 141, $\:\beta\:$ = −0.47, df = 121.29, *p* = 0.051), but was significantly and negatively associated with the proportion of the diet composed of aquatic arthropods calculated via occurrence (PO: *n* = 141, $\:\beta\:$ = −0.83, df = 125.62, *p* = 0.001). Nestlings with higher dietary diversity on day 12 were significantly more likely to survive to fledging (*n* = 137, with 26 dying and 111 fledging, $\:\beta\:$ = 1.14, *p* = 0.001, Fig. 2). Fledge success was unrelated to the proportion of aquatic arthropods in day 12 nestlings’ diets (*n* = 137, with 26 dying and 111 fledging; PRA: $\:\beta\:$ = −0.49, *p* = 0.12, PO: $\:\beta\:$ = −0.51, *p* = 0.12).

Day 15 nestling results closely matched those of day 12 nestlings. In day 15 nestlings, mass was unrelated to dietary diversity (*n* = 53, $\:\beta\:$ = 0.01, df = 48.42, *p* = 0.96) or the proportion of the diet composed of aquatic arthropods calculated via relative abundance (PRA: *n* = 53, $\:\beta\:$ = −0.13, df = 50.34, *p* = 0.59), but was significantly and negatively associated with the proportion of the diet composed of aquatic arthropods calculated via occurrence (PO: *n* = 53, $\:\beta\:$ = −0.66, df = 47.76, *p* = 0.006). Nestlings with higher dietary diversity on day 15 were significantly more likely to survive to fledging (*n* = 53, with 6 dying and 47 fledging, $\:\beta\:$ = 1.23, *p* = 0.04). Fledge success was unrelated to the proportion of aquatic arthropods in day 15 nestlings’ diets (*n* = 53, with 6 dying and 47 fledging; PRA: $\:\beta\:$ = −0.13, *p* = 0.78; PO: $\:\beta\:$ = −0.27, *p* = 0.50).

### Predictors of diet composition in nestlings

None of the measured phenotypic traits of adult females predicted the diets of their nestlings (*n* = 210, Table S3, Table S4, Table S5). Overall, site had a limited effect on nestling dietary diversity (*n* = 210, Table S3). In the full LMM, nestlings at Unit 4 had higher dietary diversity than at the other sites (Table S3). A post hoc Type III Analysis of Variance test on the model of adult phenotype and nestling dietary diversity showed that site was a nearly significant predictor (*p* = 0.08). However, post hoc pairwise comparisons showed that none of the sites differed significantly in nestling dietary diversity. In terms of aquatic arthropod diet content, site predicted neither the proportion of diets composed of aquatic arthropods calculated via relative abundance, nor the proportion of diets composed of aquatic arthropods calculated via occurrence (Table S4, Table S5).

**Fig. 3:**
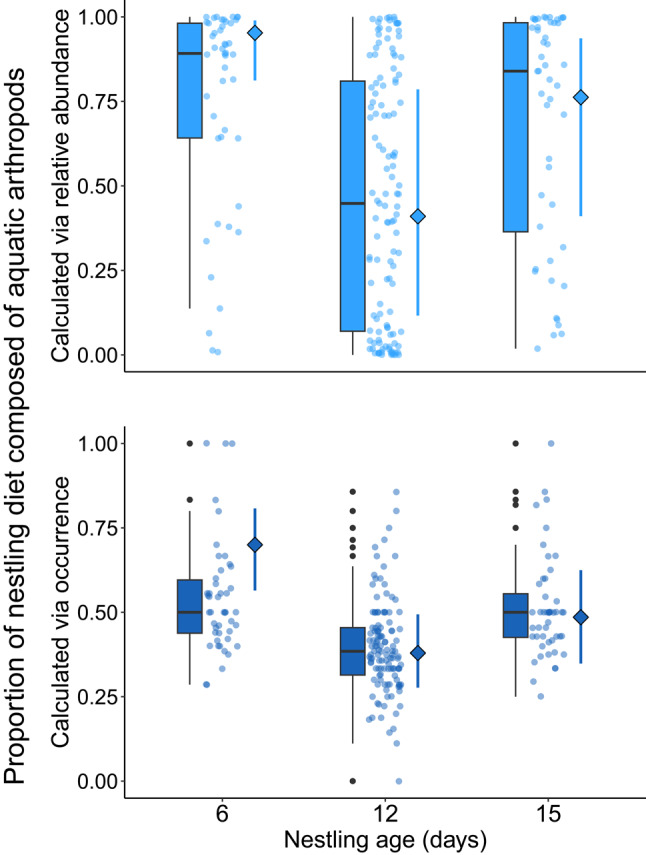
Proportion of the diet composed of aquatic arthropods, calculated via relative abundance (top panel) and occurrence (bottom panel), and nestling age (measured in days). The boxes show the minimum, first quartile, median, third quartile, and maximum values for the proportion of the nestling diet composed of aquatic arthropods for each nestling age. The points show the raw data from individual samples, and the diamonds and lines show the means and 95% confidence intervals as predicted by the model

Nestling age was strongly associated with the proportion of the diet composed of aquatic arthropods (Fig. 3), as revealed via the full LMMs with proportion aquatic arthropods calculated via relative abundance and occurrence (Table S4, Table S5) and post hoc Type III Analysis of Variance tests (PRA: age, *p* < 0.0001, PO: age, *p* < 0.0001). Day 6 nestlings had significantly higher proportions of their diets composed of aquatic arthropods than day 12 nestlings (PRA: post hoc pairwise comparison, *p* < 0.0001, Fig. 3a, PO: post hoc pairwise comparison, *p* < 0.0001, Fig. 3b), but did not have significantly different proportions of their diets composed of aquatic arthropods than day 15 nestlings (PRA: post hoc pairwise comparison, *p* = 0.06, Fig. 3a, PO: post hoc pairwise comparison, *p* = 0.06, Fig. 3b). Day 12 nestlings had significantly lower proportions of their diets composed of aquatic arthropods, calculated via relative abundance, than day 15 nestlings (PRA: post hoc pairwise comparison, *p* = 0.02, Fig. 3a). However, the proportion of aquatic arthropods in the diet calculated via occurrence did not differ between day 12 and day 15 nestlings (PO: post hoc comparison, *p* = 0.23, Fig. 3b).

### Predictors of diet composition in adults

**Fig. 4:**
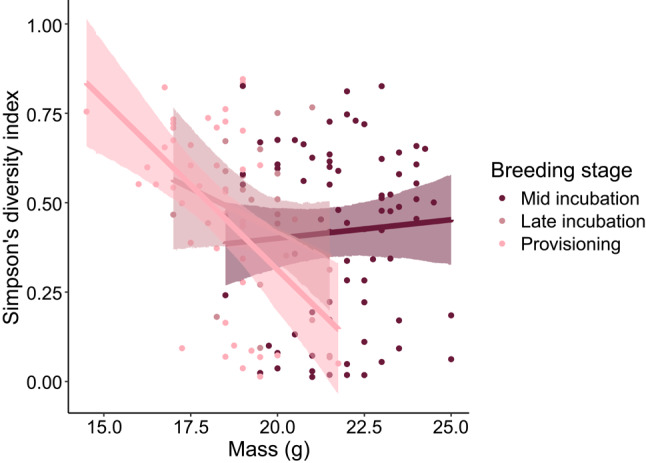
Relationship between mass (g) and Simpson’s diversity index of diet in adult female tree swallows across three breeding stages. The pink and red lines show the predicted relationship from the LMM, and the shaded pink and red areas show the 95% confidence intervals of the predictions. Predictions were made with site held constant as Unit 2. The pink and red points show raw data of individual sample

Adult female dietary diversity was predicted by mass and an interaction between mass and breeding stage (*n* = 142, Table S6, Fig. 4, post hoc Type III Analysis of Variance test, mass *p* = 0.02, mass*breeding stage *p* = 0.01). During mid and late incubation, there was no relationship between mass and dietary diversity (mid incubation: $\:\beta\:$ = 0.005, 95% confidence interval [−0.03, 0.04], late incubation: $\:\beta\:$ = −0.06, 95% confidence interval [−0.15, 0.03], Fig. 4), but lighter females had more diverse diets during provisioning ($\:\beta\:$ = −0.10, 95% confidence interval [−0.16, −0.04], Fig. 4). However, adult female dietary diversity was not predicted by mass loss between mid incubation and provisioning (*n* = 45, Table S7). In contrast to dietary diversity, neither female mass nor female mass loss predicted the proportion of the diets composed of aquatic arthropods (mass: PRA: Table S8, PO: Table S9; mass loss: PRA: Table S10, PO: Table S11). There were no significant relationships between female wing length and dietary diversity (Table S6) or the proportion of the diets composed of aquatic arthropods (PRA: Table S8, PO: Table S9).

The dietary diversity of adult females did not differ across sites (Table S6, post hoc Type III Analysis of Variance test, site *p* = 0.44), but site did predict the proportion of their diets composed of aquatic arthropods calculated via relative abundance (*n* = 142, Table S8, post hoc Type III Analysis of Variance test, site *p* = 0.0009). Females from Unit 4 had significantly lower proportions of their diets composed of aquatic arthropods than females from Turkey Hill (post hoc pairwise comparison, *p* = 0.0004), Unit 1 (post hoc pairwise comparison, *p* = 0.02), and Unit 2 (post hoc pairwise comparison, *p* = 0.009). When the proportion of the diet composed of aquatic arthropods was calculated via occurrence, there were no significant predictors of the proportion of the diet composed of aquatic arthropods (Table S9).

### Diet composition in adults versus nestlings

After examining adult and nestling diets separately, we tested whether diet was different in adult females, adult males, and nestlings. These groups did not differ in their dietary diversity or in the proportion of their diets composed of aquatic arthropods measured via occurrence (*n* = 331, Table S12). In the full LMM for relative abundance, females showed a significantly higher proportion of their diets composed of aquatic arthropods than nestlings (*n* = 331, Table S12). However, a post hoc Type III Analysis of Variance test found that adult males, adult females, and nestlings were not significantly different in the proportion of their diets composed of aquatic arthropods (PRA: *p* = 0.12). Post hoc pairwise comparisons also revealed that none of the groups differed in diet aquatic arthropod content (PRA: adult females and adult males, *p* = 0.24; adult females and nestlings, *p* = 0.10; adult males and nestlings, *p* = 1.00).

## Discussion

### Diet, nestling mass, and fledging success

Our results suggest that dietary diversity may play an important, and previously unrecognized, role in tree swallow reproductive success. Nestlings with more diverse diets on both day 12 and day 15 had a higher probability of fledging. To our knowledge, this is the first time that dietary diversity has been linked to the reproductive success of a wild passerine. Furthermore, dietary diversity did not predict nestling mass on either day 12 or day 15. This suggests that the relationship we see between diversity and fledge success is likely not because dietary diversity is a proxy for the amount of food received, but rather, it holds specific value for the health of these birds. Tree swallow nestlings may rely on diverse diets to provide balanced inputs of macronutrients and micronutrients important for overall health. Future work should explore how diverse diets balance nutritional needs.

Surprisingly, we found no evidence for a relationship between the proportion of nestling diet composed of aquatic arthropods and fledging success, and nestlings with higher proportions of aquatic arthropods in their diets (calculated via occurrence) on days 12 and 15 had lower body mass. Previous work on this tree swallow study population has found a strong relationship between annual variation in the availability of aerial aquatic insects and reproductive success (Twining et al. 2018b). Experimental manipulations of HUFA dietary content in lab-reared nestling tree swallows also confirmed their importance for nestling growth (Twining et al. 2016b). Furthermore, Elgin (2019) suggested that a greater ratio of aquatic to terrestrial insects in the diet positively predicted nestling mass in another population of tree swallows.

It is possible that diets composed of higher proportions of aquatic arthropods, calculated via occurrence, might indicate that tree swallows cannot find their preferred aquatic insect prey species. In this case, they might still seek to find aquatic prey due to its nutritional value but be forced to expand to foraging for numerous additional, less preferred aquatic families that might have less favorable nutritional value. If this is the case, then increased proportions of aquatic arthropods calculated via occurrence may be a sign of prey limitation, as suggested by Hoenig et al. (2022). This could explain the lower body masses of nestlings with diets composed of higher proportions of aquatic arthropods calculated via occurrence.

One possible explanation for the lack of relationship between the proportion of the diet composed of aquatic arthropods (calculated via relative abundance) and fitness-related metrics is that there may be a baseline level of HUFAs that tree swallow nestlings need to develop successfully, and beyond that level any additional HUFA input will have no appreciable effect on reproductive success. In alignment with this study’s findings, work on a tree swallow population in Saskatchewan, Canada found that stable isotopes in the blood of nestlings (which differ depending on the relative amount of aquatic versus terrestrial prey consumed) were not related to body mass or condition (Michelson et al. 2018). Michelson et al. (2018) cover only 2012–2013 and this study covers only 2019. During these times aquatic arthropod availability may have been high at the study sites such that all birds were able to acquire enough HUFA-rich diet items for their nestlings. Indeed, three of the four most common arthropod families in tree swallow diets in this study were aquatic (Limoniidae, Chironomidae, and Culicidae), suggesting that tree swallows took in large quantities of these HUFA-rich insects in the year of this study.

Furthermore, due to limitations of DNA metabarcoding, this study could not characterize the biomass of aquatic arthropods in diets and relies on their relative abundance. Thus, even if nestlings have diets composed of lower proportions of aquatic arthropods, they may still be getting enough biomass to support their needs. One major strength of Twining et al. (2018b) is that researchers could characterize the available insect biomass; however, they could not characterize individual nestling diets. Future work could combine both local aerial insect biomass measurements with fecal DNA metabarcoding to gain a fuller perspective on the relationship between aquatic insect availability, aerial insectivore prey consumption, and aerial insectivore reproductive outcomes. Additionally, future work should prioritize identifying the threshold level of HUFAs needed for successful aerial insectivore development.

### Predictors of diet composition in nestlings and adults

We found that the relationship between adult female tree swallow mass and dietary diversity changed over the course of the breeding season. During mid and late incubation, mass was unrelated to dietary diversity, but during provisioning, adult females with higher dietary diversity had lower mass. (However, in a complementary analysis, we found that mass change between captures had no association with dietary diversity.) The relationship between mass and dietary diversity during provisioning could be explained by the birds’ adaptive mass loss, in which females lose mass to forage more efficiently during the provisioning period (Boyle et al. 2012). One possibility is that females with lower body masses during provisioning move more, and this greater movement might lead them to gather food from more disparate habitats leading to higher dietary diversity. Other than the relationship between female mass and dietary diversity, we found no significant relationships between an adult female’s phenotypic traits and her diet or her nestlings’ diets.

Site explained some variation in diet content in tree swallows. As we predicted, there was some evidence that adult females at Unit 4, which is the driest of our four field sites, had lower proportions of their diets composed of aquatic arthropods. Contrary to our expectation, there was some evidence that site predicted nestling dietary diversity, with Unit 4 nestlings having higher dietary diversity than other sites. These results suggest that either there is a greater diversity of arthropods available at Unit 4, or that parents are feeding their nestlings more prey types there which could result from foraging over greater distances or from active selection of specific prey items. Another possibility is that Unit 4 birds could be diversifying their diets due to lack of their preferred aquatic insects in the environment. The land cover around tree swallow breeding sites likely affects their diets, though this work did not have enough replicates of different habitat types to fully explore how these habitats influence diet and the implications this could have for tree swallow health and fitness. Future research should explore how specific land cover features (agricultural land, water features, etc.) affect the diets of tree swallows and other generalist animals, focusing especially on the consequences for dietary diversity and macronutrient content in the diet.

Our results show that adults feed their nestlings differing proportions of aquatic arthropods versus terrestrial arthropods over the course of their development. Specifically, adults feed their nestlings especially high proportions of aquatic arthropods on day 6 of the nestling period, which is just prior to the start of the nestlings’ exponential growth period (Zach and Mayoh 1982; Twining et al. 2018b). There are many potential explanations for this pattern. One possibility is that HUFAs have greater importance during certain stages of development, so adults seek out HUFA-rich aquatic arthropods when they are foraging for their nestlings on those days. A second, and not mutually exclusive, possibility is that aquatic arthropod abundance in the environment happens to peak during certain stages of nestling development (for example, see Twining et al. 2018b for insect peaks measured at one site in Tompkins County, NY), and so these HUFA-rich arthropods are simply more widely available to adults foraging for their nestlings. Interestingly, the proportion of the diet composed of aquatic arthropods dropped in day 12 nestlings and increased again in day 15 nestlings. This pattern may also be related to variation in availability of aquatic arthopods over the course of development, and merits further investigation in future studies.

### Diet composition in adults versus nestlings

Contrary to our prediction, we found no evidence that nestlings had diets composed of higher proportions of aquatic arthropods than adults, and in fact, we found limited evidence that females had diets composed of higher proportions of aquatic arthropods than nestlings. Michelson et al. (2018) found that adult tree swallows in Saskatchewan, Canada had higher proportions of aquatic insect orders in their diets than nestlings. Adult and nestling diet similarities and differences could vary on a year-to-year and site-to-site basis, and more work should compare these groups’ diets to investigate whether there is stronger evidence that females have diets composed of higher proportions of aquatic arthropods than nestlings.

There was also no evidence that adult females had different dietary diversities or proportions of aquatic arthropods in their diets than adult males. This suggests that, as we predicted, there are no differences in dietary diversity or HUFA needs across the sexes. However, we could only compare the sexes’ diets during provisioning because that is the only breeding stage when we captured both females and males. Future studies could seek to collect male samples during other stages of breeding to further explore differences in male versus female diets across the breeding season. This type of comparison would be especially fascinating during the egg laying and incubation periods, when females may have different energetic needs.

### Relative abundance of reads versus occurrence of reads

In this study, we used both relative abundance of reads and occurrence of reads to summarize our diet metabarcoding results. Some argue that occurrence is a more conservative option than relative abundance because relative abundance can suffer from recovery bias and digestion bias (Deagle et al. 2019). However, others posit that occurrence is also biased because it considers rare taxa as equal to common taxa in an animal’s diet, potentially overinflating the importance of rare taxa – including those that might be environmental contaminants (Deagle et al. 2019).

Several characteristics of our study suggest that it may be less subject to biases associated with measurements of relative read abundance. Deagle et al. (2019) suggest that using relative abundance may be especially appropriate when a number of taxa occur in each fecal sample, and the same taxa occur across many samples; this is the case for our data, as we have a number of taxa in each sample and many families shared across samples. Furthermore, Piñol et al. (2019) found that, when the number of species in a mixture is higher, the probability of metabarcoding results accurately reflecting their relative abundance is also higher, and they specifically mention insectivorous birds as a candidate for good dietary quantification. Verkuil et al. (2022) used camera traps to monitor what pied flycatchers (*Ficedula hypoleuca*), another insectivorous songbird, fed their nestlings; they then used DNA metabarcoding of nestling feces to confirm that relative read abundance reflected relative biomass of dietary items. Finally, in a test of multiple primer pairs, Piñol et al. (2019) found that the primer pair we use in this study (BF2 and BR2) worked better than others for quantitative DNA metabarcoding.

Recently, Schmiedová et al. (2022) used primers BF2 and BR2 for diet metabarcoding in another aerial insectivore, barn swallows (*Hirundo rustica*), and reported their results in terms of relative read abundance. In a second recent study that used fecal DNA metabarcoding to measure aerial insectivore diet, Arazmi et al. (2025) reported results using both relative read abundance and frequency of occurrence. Future studies should report both relative read abundance and occurrence results, when possible; this gives readers more information to help interpret results.

### Limitations and future opportunities

This study occurred over the course of a single breeding season (2019), and across four sites that were 10 km or less apart. Therefore, the patterns discovered merit further investigation across multiple breeding seasons, and across multiple locations, as arthropod availability may vary greatly across time and space. Furthermore, fecal DNA metabarcoding has inherent limitations; it provides only a snapshot of what animals are eating within a day or two of sample collection (e.g., Thuo et al. 2019), and issues like primer bias (Elbrecht and Leese 2015) and differential digestion of DNA from different food sources through the digestive tract (Deagle and Tollit 2007) can lead to biased results. Nevertheless, all diet sampling methods result in biases, and fecal sample DNA metabarcoding remains an excellent non-invasive method for collecting large numbers of diet samples, as we have done in this study. We hope this serves as a starting point for future diet metabarcoding studies; multi-year studies that cover larger geographic areas would be of particular use in elucidating the relationship between diet composition and fitness in generalist animals on a broader scale.

## Conclusion

Our results suggest that dietary diversity may be important to a generalist bird’s reproductive success. More broadly, dietary diversity could be an underappreciated dietary trait that merits further attention as aerial insectivore conservation efforts take flight. From an anthropogenic perspective, the link between a diverse diet and health seems obvious, but its importance has been less clear for generalist non-human animals. Future research should continue to explore the importance of dietary diversity for generalists and determine whether diets have “keystone” components that can make or break reproductive success. This is especially important for declining groups like aerial insectivores (Rosenberg et al. 2019). Conservation efforts could be further buoyed by recognition of ecosystem services (Daily 1997) provided by aerial insectivores such as pest control; for example, we found that tree swallows’ diets often contained mosquitoes (Culicidae). Additional work could explore whether different species of aerial insectivores control pest insects, and the value this has for human populations. Moving forward, fecal DNA metabarcoding, especially in combination with other dietary characterization techniques (Hoenig et al. 2022), remains a promising avenue for identifying generalists’ diets and providing further insight into the importance of dietary diversity across taxa.

## Supplementary Information

Below is the link to the electronic supplementary material.

Supplementary Material 1

## Data Availability

Raw sequencing data are uploaded to NCBI’s Sequence Read Archive (Bioproject: PRJNA884756). Output from the AMPtk pipeline, metadata, and all code to produce the analyses for this paper are available on GitHub at https://github.com/juehling/tres_coi_analysis_2019_ms.

## References

[CR1] Arazmi FN, Ismail NA, Daud UNS, Mansor MS (2025) DNA metabarcoding reveals distinct trophic niches among sympatric aerial insectivores (Family: Apodidae and Hirundinidae) in central Peninsular Malaysia. Nat Conserv 59:207–229. 10.3897/natureconservation.59.152167

[CR2] Bates D, Mächler M, Bolker BM, Walker SC (2015) Fitting linear mixed-effects models using lme4. J Stat Softw 67:1–48. 10.18637/jss.v067.i01

[CR3] Beck ML, Hopkins WA, Jackson BP (2013) Spatial and temporal variation in the diet of tree swallows: implications for trace-element exposure after habitat remediation. Arch Environ Contam Toxicol 65:575–587. 10.1007/s00244-013-9913-5 23695717 10.1007/s00244-013-9913-5

[CR4] Belgrad BA, Griffen BD (2016) The influence of diet composition on fitness of the blue crab, *Callinectes sapidus*. PLoS One 11:e0145481. 10.1371/journal.pone.0145481 26784581 10.1371/journal.pone.0145481 PMC4718683

[CR5] Bellavance V, Bélisle M, Savage J (2018) Influence of agricultural intensification on prey availability and nestling diet in tree swallows (*Tachycineta bicolor*). Can J Zool 96:1053–1065. 10.1139/cjz-2017-0229

[CR103] Bolker B, Warnes G, Lumley T (2022) gtools: Various R Programming Tools. R package version 3.9.4. https://CRAN.R-project.org/package=gtools

[CR6] Bolnick DI, Svanbäck R, Araújo MS, Persson L (2007) Comparative support for the niche variation hypothesis that more generalized populations also are more heterogeneous. Proc Natl Acad Sci USA 104:10075–10079. 10.1073/pnas.0703743104 17537912 10.1073/pnas.0703743104 PMC1891261

[CR7] Bolnick DI, Snowberg LK, Hirsch PE (2014) Individuals’ diet diversity influences gut microbial diversity in two freshwater fish (threespine stickleback and Eurasian perch). Ecol Lett 17:979–987. 10.1111/ele.12301 24847735 10.1111/ele.12301 PMC4084827

[CR8] Boyle WA, Winkler DW, Guglielmo CG (2012) Rapid loss of fat but not lean mass prior to chick provisioning supports the flight efficiency hypothesis in tree swallows. Funct Ecol 26:895–903. 10.1111/j.1365-2435.2012.01997.x

[CR9] Brenna JT, Carlson SE (2014) Docosahexaenoic acid and human brain development: evidence that a dietary supply is needed for optimal development. J Hum Evol 77:99–106. 10.1016/j.jhevol.2014.02.017 24780861 10.1016/j.jhevol.2014.02.017

[CR10] Bumelis KH, Cadman MD, Hobson KA (2022) Endogenous biomarkers reveal diet partitioning among three sympatric species of swallows. Ornithology 139:ukab078. 10.1093/ornithology/ukab078

[CR11] Callahan BJ, McMurdie PJ, Rosen MJ et al (2016) DADA2: high-resolution sample inference from Illumina amplicon data. Nat Methods 13:581–583 27214047 10.1038/nmeth.3869 PMC4927377

[CR12] Christensen-Dalsgaard S, May R, Barrett R et al (2018) Prevailing weather conditions and diet composition affect chick growth and survival in the black-legged kittiwake. Mar Ecol Prog Ser 604:237–249. 10.3354/meps12744

[CR13] Cook JG, Johnson BK, Cook RC et al (2004) Effects of summer-autumn nutrition and parturition date on reproduction and survival of elk. Wildl Monogr 155:1–61

[CR14] Cruz-Rivera E, Hay ME (2000) The effects of diet mixing on consumer fitness: macroalgae, epiphytes, and animal matter as food for marine amphipods. Oecologia 123:252–264. 10.1007/s004420051012 28308730 10.1007/s004420051012

[CR15] Daily GC (ed) (1997) Nature’s Services: Societal Dependence On Natural Ecosystems. Island

[CR16] Deagle BE, Tollit DJ (2007) Quantitative analysis of prey DNA in pinniped faeces: potential to estimate diet composition? Conserv Genet 8:743–747. 10.1007/s10592-006-9197-7

[CR17] Deagle BE, Thomas AC, McInnes JC et al (2019) Counting with DNA in metabarcoding studies: how should we convert sequence reads to dietary data? Mol Ecol 28:391–406. 10.1111/mec.14734 29858539 10.1111/mec.14734 PMC6905394

[CR18] DeMott WR (1998) Utilization of a cyanobacterium and a phosphorus-deficient green alga as complementary resources by daphnids. Ecology 79:2463–2481. 10.1890/0012-9658(1998)079[2463:UOACAA]2.0.CO;2

[CR19] Elbrecht V, Leese F (2015) Can DNA-based ecosystem assessments quantify species abundance? Testing primer bias and biomass—sequence relationships with an innovative metabarcoding protocol. PLOS ONE 10:e0130324. 10.1371/journal.pone.0130324 26154168 10.1371/journal.pone.0130324 PMC4496048

[CR20] Elgin AS (2019) Conserving prairie ponds for swallows: Tree swallow (*Tachycineta bicolor*) foraging and nestling diet quality in prairie agroecosystems. Master’s Thesis, University of Saskatchewan

[CR22] Emlen JM (1966) The role of time and energy in food preference. Am Nat 100:611–617. 10.1086/282455

[CR23] Forsman AM, Hoenig BD, Gaspar SA et al (2022) Evaluating the impacts of metabarcoding primer selection on DNA characterization of diet in an aerial insectivore, the Purple Martin. Ornithology 139:ukab075. 10.1093/ornithology/ukab075

[CR24] Griffen BD (2014) Linking individual diet variation and fecundity in an omnivorous marine consumer. Oecologia 174:121–130. 10.1007/s00442-013-2751-3 23996228 10.1007/s00442-013-2751-3

[CR25] Guillod N, Arlettaz R, Jacot A (2016) Impact of spatial variation of a crucial prey, the molecricket, on hoopoe territory occupancy and reproduction. J Avian Biol 47:697–705. 10.1111/jav.00990

[CR26] Hall LA, De La Cruz SEW, Woo I et al (2021) Age- and sex‐related dietary specialization facilitate seasonal resource partitioning in a migratory shorebird. Ecol Evol 11:1866–1876. 10.1002/ece3.7175 33614009 10.1002/ece3.7175 PMC7882968

[CR27] Harrison SJ, Raubenheimer D, Simpson SJ et al (2014) Towards a synthesis of frameworks in nutritional ecology: interacting effects of protein, carbohydrate and phosphorus on field cricket fitness. Proc R Soc Lond B Biol Sci 281:20140539. 10.1098/rspb.2014.0539 10.1098/rspb.2014.0539 PMC4150310 25143029

[CR28] Heiman ML, Greenway FL (2016) A healthy gastrointestinal microbiome is dependent on dietary diversity. Mol Metab 5:317–320. 10.1016/j.molmet.2016.02.005 27110483 10.1016/j.molmet.2016.02.005 PMC4837298

[CR29] Hixson SM, Sharma B, Kainz MJ et al (2015) Production, distribution, and abundance of long-chain omega-3 polyunsaturated fatty acids: a fundamental dichotomy between freshwater and terrestrial ecosystems. Environ Rev 23:414–424. 10.1139/er-2015-0029

[CR30] Hoenig BD, Trevelline BK, Nuttle T, Porter BA (2021) Dietary DNA metabarcoding reveals seasonal trophic changes among three syntopic freshwater trout species. Freshw Biol 66:509–523. 10.1111/fwb.13656

[CR31] Hoenig BD, Trevelline BK, Kautz A et al (2022) Two is better than one: coupling DNA metabarcoding and stable isotope analysis improves dietary characterizations for a riparian-obligate, migratory songbird. Mol Ecol mec.16688. 10.1111/mec.16688 10.1111/mec.16688 36089910

[CR32] Jump DB (2002) The biochemistry of n-3 polyunsaturated fatty acids. J Biol Chem 277:8755–8758. 10.1074/jbc.R100062200 11748246 10.1074/jbc.R100062200

[CR33] Karasov WH, del Martinez C (2007) Physiological ecology: how animals process energy, nutrients and toxins. Princeton Univ. Press

[CR34] Kartzinel TR, Hsing JC, Musili PM et al (2019) Covariation of diet and gut microbiome in African megafauna. Proc Natl Acad Sci U S A 116:23588–23593. 10.1073/pnas.1905666116 31685619 10.1073/pnas.1905666116 PMC6876249

[CR101] Kim BR, Shin J, Guevarra RB, Lee JH, Kim DW, Seol KH, Isaacson RE (2017) Deciphering Diversity Indices for a Better Understanding of Microbial Communities. J Microbio Biotechnol 27(12):2089–2093. 10.4014/jmb.1709.09027 10.4014/jmb.1709.09027 29032640

[CR35] Kim TN, Bukhman YV, Jusino MA, Scully ED, Spiesman BJ, Gratton C (2022) Using high-throughput amplicon sequencing to determine diet of generalist lady beetles in agricultural landscapes. Biol Control 170:104920

[CR36] Kumar RAM, Ramakrishna Rao T (1999) Demographic responses of adult *Mesocyclops thermocyclopoides* (Copepoda, Cyclopoida) to different plant and animal diets. Freshw Biol 42:487–501. 10.1046/j.1365-2427.1999.00485.x

[CR37] Kutzer MAM, Kurtz J, Armitage SAO (2018) Genotype and diet affect resistance, survival, and fecundity but not fecundity tolerance. J Evol Biol 31:159–171. 10.1111/jeb.13211 29150962 10.1111/jeb.13211

[CR38] Lefcheck JS, Whalen MA, Davenport TM et al (2013) Physiological effects of diet mixing on consumer fitness: a meta-analysis. Ecology 94:565–572. 10.1890/12-0192.1 23687882 10.1890/12-0192.1

[CR104] Lenth R (2024) emmeans: Estimated Marginal Means, aka Least-Squares Means. R package version 1.10.1. https://CRAN.R-project.org/package=emmeans

[CR39] Lourenço R, Delgado M, Campioni L et al (2015) Evaluating the influence of diet-related variables on breeding performance and home range behaviour of a top predator. Popul Ecol 57:625–636. 10.1007/s10144-015-0506-1

[CR40] MacArthur RH, Pianka ER (1966) On Optimal Use of a Patchy Environment. Am Nat 100:603–609. 10.1086/282454

[CR41] Magalhães de Oliveira HF, Camargo NF, Hemprich-Bennett DR et al (2020) Wing morphology predicts individual niche specialization in *Pteronotus mesoamericanus* (Mammalia: Chiroptera). PLoS One 15:e0232601. 10.1371/journal.pone.0232601 32392221 10.1371/journal.pone.0232601 PMC7213686

[CR42] Marchetti K, Price T, Richman A (1995) Correlates of Wing Morphology with Foraging Behaviour and Migration Distance in the Genus *Phylloscopus*. J Avian Biol 26:177. 10.2307/3677316

[CR43] Margalida A, Benítez JR, Sánchez-Zapata JA et al (2012) Long-term relationship between diet breadth and breeding success in a declining population of Egyptian Vultures *Neophron percnopterus*. Ibis 154:184–188. 10.1111/j.1474-919X.2011.01189.x

[CR44] McCarty JP, Winkler DW (1999) Foraging Ecology and Diet Selectivity of Tree Swallows Feeding Nestlings. Condor 101:246–254

[CR45] McMurdie PJ, Holmes S (2013) Phyloseq: an R package for reproducible interactive analysis and graphics of microbiome census data. PLoS One 8:e61217. 10.1371/journal.pone.0061217 23630581 10.1371/journal.pone.0061217 PMC3632530

[CR46] Michelson CI, Clark RG, Morrissey CA (2018) Agricultural land cover does not affect the diet of tree swallows in wetland-dominated habitats. Condor 120:751–764. 10.1650/CONDOR-18-16.1

[CR47] Milá B, Wayne RK, Smith TB (2008) Ecomorphology of migratory and sedentary populations of the yellow-rumped warbler (*Dendroica coronata*). Condor 110:335–344. 10.1525/cond.2008.8396

[CR48] Nebel S, Mills A, McCracken JD, Taylor PD (2010) Declines of aerial insectivores in North America follow a geographic gradient. Avian Conserv Ecol 5:1. 10.5751/ACE-00391-050201

[CR100] Oksanen J, Simpson G, Blanchet F, Kindt R, Legendre P, Minchin P, O'Hara R, Solymos P, Stevens M, Szoecs E, Wagner H, Barbour M, Bedward M, Bolker B, Borcard D, Carvalho G, Chirico M, De Caceres M, Durand S, Evangelista H, FitzJohn R, Friendly M, Furneaux B, Hannigan G, Hill M, Lahti L, McGlinn D, Ouellette M, Ribeiro Cunha E, Smith T, Stier A, Ter Braak C, Weedon J (2022) vegan: Community Ecology Package. R package version 2.6.4. https://CRAN.R-project.org/package=vegan

[CR49] Palmer JM, Jusino MA, Banik MT, Lindner DL (2018) Non-biological synthetic spike-in controls and the AMPtk software pipeline improve mycobiome data. PeerJ 6:e4925. 10.7717/peerj.4925 29868296 10.7717/peerj.4925 PMC5978393

[CR50] Piñol J, Senar MA, Symondson WOC (2019) The choice of universal primers and the characteristics of the species mixture determine when DNA metabarcoding can be quantitative. Mol Ecol 28:407–419. 10.1111/mec.14776 29939447 10.1111/mec.14776

[CR51] R Core Team (2023) R: A Language and Environment for Stastical Computing. R Foundation for Statistical Computing, Vienna, Austria

[CR52] Raubenheimer D, Simpson SJ (2018) Nutritional ecology and foraging theory. Curr Opin Insect Sci 27:38–45. 10.1016/j.cois.2018.02.002 30025633 10.1016/j.cois.2018.02.002

[CR53] Raubenheimer D, Simpson SJ, Mayntz D (2009) Nutrition, ecology and nutritional ecology: toward an integrated framework. Funct Ecol 23:4–16. 10.1111/j.1365-2435.2009.01522.x

[CR54] Resano-Mayor J, Hernández-Matías A, Real J et al (2014) Multi-scale effects of nestling diet on breeding performance in a terrestrial top predator inferred from stable isotope analysis. PLoS One 9:e95320. 10.1371/journal.pone.0095320 24743233 10.1371/journal.pone.0095320 PMC3990674

[CR55] Rosenberg KV, Dokter AM, Blancher PJ et al (2019) Decline of the North American avifauna. Science 366:120–124. 10.1126/science.aaw1313 31604313 10.1126/science.aaw1313

[CR56] Schleuter D, Eckmann R (2008) Generalist versus specialist: the performances of perch and ruffe in a lake of low productivity. Ecol Freshw Fish 17:86–99. 10.1111/j.1600-0633.2007.00262.x

[CR57] Schmiedová L, Tomášek O, Pinkasová H et al (2022) Variation in diet composition and its relation to gut microbiota in a passerine bird. Sci Rep 12:3787. 10.1038/s41598-022-07672-9 35260644 10.1038/s41598-022-07672-9 PMC8904835

[CR58] Serrano-Davies E, Sanz JJ (2017) Habitat structure modulates nestling diet composition and fitness of Blue Tits *Cyanistes caeruleus* in the Mediterranean region. Bird Study 64:295–305. 10.1080/00063657.2017.1357678

[CR59] Shaner PL, Chen Y, Hsu Y (2021) Niche–trait relationships at individual and population level in three co-occurring passerine species. Ecol Evol 11:7378–7389. 10.1002/ece3.7569 34188820 10.1002/ece3.7569 PMC8216981

[CR102] Simpson EH (1949) Measurement of Diversity. Nature 163:688. 10.1038/163688a0

[CR60] Smith JA, Baumgartner LJ, Suthers IM, Taylor MD (2011) Generalist niche, specialist strategy: the diet of an Australian percichthyid. J Fish Biol 78:1183–1199. 10.1111/j.1095-8649.2011.02926.x 21463314 10.1111/j.1095-8649.2011.02926.x

[CR61] Smith AC, Hudson M-A, Downes CM, Francis CM (2015) Change points in the population trends of aerial-insectivorous birds in North America: synchronized in time across species and regions. PLoS One 10:e0130768. 10.1371/journal.pone.0130768 26147572 10.1371/journal.pone.0130768 PMC4493114

[CR62] Solís-García IA, Ceballos-Luna O, Cortazar-Murillo EM, Desgarennes D, Garay-Serrano E, Patiño-Conde V, Guevara-Avendaño E, Méndez-Bravo A, Reverchon F (2021) *Phytophthora* root rot modifies the composition of the avocado rhizosphere microbiome and increases the abundance of opportunistic fungal pathogens. Front Microbiol 11:574110 33510714 10.3389/fmicb.2020.574110 PMC7835518

[CR63] Spiller KJ, Dettmers R (2019) Evidence for multiple drivers of aerial insectivore declines in North America. Condor 121:duz010. 10.1093/condor/duz010

[CR64] Terraube J, Arroyo B, Madders M, Mougeot F (2011) Diet specialisation and foraging efficiency under fluctuating vole abundance: a comparison between generalist and specialist avian predators. Oikos 120:234–244. 10.1111/j.1600-0706.2010.18554.x

[CR65] Thuo D, Furlan E, Broekhuis F et al (2019) Food from faeces: evaluating the efficacy of scat DNA metabarcoding in dietary analyses. PLoS One 14:e0225805. 10.1371/journal.pone.0225805 31851671 10.1371/journal.pone.0225805 PMC6980833

[CR66] Tiede J, Scherber C, Mutschler J et al (2017) Gut microbiomes of mobile predators vary with landscape context and species identity. Ecol Evol 7:8545–8557. 10.1002/ece3.3390 29075470 10.1002/ece3.3390 PMC5648672

[CR67] Turnbaugh PJ, Gordon JI (2009) The core gut microbiome, energy balance and obesity. J Physiol 587:4153–4158. 10.1113/jphysiol.2009.174136 19491241 10.1113/jphysiol.2009.174136 PMC2754355

[CR68] Twining CW, Brenna JT, Hairston NG, Flecker AS (2016a) Highly unsaturated fatty acids in nature: what we know and what we need to learn. Oikos 125:749–760. 10.1111/oik.02910

[CR69] Twining CW, Brenna JT, Lawrence P et al (2016b) Omega-3 long-chain polyunsaturated fatty acids support aerial insectivore performance more than food quantity. Proc Natl Acad Sci U S A 113:10920–10925. 10.1073/pnas.1603998113 27638210 10.1073/pnas.1603998113 PMC5047183

[CR70] Twining CW, Lawrence P, Winkler DW et al (2018a) Conversion efficiency of alpha linolenic acid to omega-3 highly unsaturated fatty acids in aerial insectivore chicks. J Exp Biol 221:jeb165373. 10.1242/jeb.165373 29217628 10.1242/jeb.165373

[CR71] Twining CW, Shipley JR, Winkler DW (2018b) Aquatic insects rich in omega-3 fatty acids drive breeding success in a widespread bird. Ecol Lett 21:1812–1820. 10.1111/ele.13156 30251301 10.1111/ele.13156

[CR72] Twining CW, Brenna JT, Lawrence P et al (2019) Aquatic and terrestrial resources are not nutritionally reciprocal for consumers. Funct Ecol 33:2042–2052. 10.1111/1365-2435.13401

[CR150] Uehling JJ (2023) Stress eating? Causes and consequences of variation in foraging behavior in a challenging world. Cornell University. https://ecommons.cornell.edu/entities/publication/0da3015c-bc1c-4eea-b7fc-02c9fff90798

[CR73] Verkuil YI, Nicolaus M, Ubels R, Dietz MW, Samplonius JM, Galema A, Kiekebos K, de Knijff P, Both C (2022) DNA metabarcoding quantifies the relative biomass of arthropod taxa in songbird diets: validation with camera-recorded diets. Ecol Evol 12:e8881. 10.1002/ece3.8881 35571761 10.1002/ece3.8881 PMC9077022

[CR74] Vitousek MN, Taff CC, Ardia DR et al (2018a) The lingering impact of stress: brief acute glucocorticoid exposure has sustained, dose-dependent effects on reproduction. Proc R Soc Lond B Biol Sci 285:20180722. 10.1098/rspb.2018.0722 10.1098/rspb.2018.0722 PMC6053934 30051820

[CR75] Vitousek MN, Taff CC, Hallinger KK et al (2018b) Hormones and Fitness: Evidence for Trade-Offs in Glucocorticoid Regulation Across Contexts. Front Ecol Evol 6:42. 10.3389/fevo.2018.00042

[CR76] Wang Y, Smith HK, Goossens E et al (2021) Diet diversity and environment determine the intestinal microbiome and bacterial pathogen load of fire salamanders. Sci Rep 11:20493. 10.1038/s41598-021-98995-6 34650115 10.1038/s41598-021-98995-6 PMC8516891

[CR77] Warton DI, Hui FKC (2011) The arcsine is asinine: the analysis of proportions in ecology. Ecology 92:3–10. 10.1890/10-0340.1 21560670 10.1890/10-0340.1

[CR78] Whitfield DP, Reid R, Haworth PF et al (2009) Diet specificity is not associated with increased reproductive performance of Golden Eagles *Aquila chrysaetos* in western Scotland. Ibis 151:255–264. 10.1111/j.1474-919X.2009.00924.x

[CR79] Winkler DW, Hallinger KK, Ardia DR et al (2020) Tree Swallow (Tachycineta bicolor), version 1.0. In: Poole AF (ed) Birds of the World. Cornell Lab of Ornithology, Ithaca, NY, USA. 10.2173/bow.treswa.01

[CR80] Woo KJ, Elliott KH, Davidson M et al (2008) Individual specialization in diet by a generalist marine predator reflects specialization in foraging behaviour. J Anim Ecol 77:1082–1091. 10.1111/j.1365-2656.2008.01429.x 18624834 10.1111/j.1365-2656.2008.01429.x

[CR81] Zach R, Mayoh KR (1982) Weight and feather growth of nestling tree swallows. Can J Zool 60:1080–1090. 10.1139/z82-149

