# Supplementary material for: Predictors and consequences of diet composition in a declining generalist aerial insectivore
Source: Oecologia. 2026 Jun 4;208(6):77. doi: 10.1007/s00442-026-05901-w (PMC13236765; doi:10.1007/s00442-026-05901-w)
Supplement: Supplementary file 1 — Supplementary Material 1 [file 442_2026_5901_MOESM1_ESM.docx]

**Online Resource 1:** Electronic Supplemental Material for “Predictors and consequences of diet composition in a declining generalist aerial insectivore”

**Submitted to**: Oecologia

**Authors**: Jennifer J. Uehling*, Conor C. Taff, Jennifer L. Houtz, Paige M. Becker, Allison S. Injaian, and Maren N. Vitousek

* Corresponding author, Email: [juehling@wcupa.edu](mailto:juehling@wcupa.edu), Telephone: 610-436-2457

**METHODS**:

*Experiment details*: As part of ongoing experimental work in the Tompkins County, NY, USA tree swallow system, two experiments were being carried out in 2019: a predator and plumage manipulation experiment, and an artificial light at night (ALAN) experiment.

The predator and plumage manipulation experiment included individuals from Unit 1 and Unit 2. This was a 2x2 factorial experiment. At the first capture (day 6-7 of incubation, “mid incubation”), females were alternately assigned to either a control or a predation treatment group within each age class. Note that the birds did not experience any treatment prior to or during the first capture, so all female fecal samples collected from Unit 1 and Unit 2 at the first capture were classified as “Control.” The females in the predation group received three simulated predation attempts by a mink (*Neovision vision*) between days 8-12 of incubation. For these simulated predation attempts, the researcher put a taxidermied mink around their hand. They then trapped the female inside her nest box and pulled her out of the nest box with the hand with the taxidermied mink. They moved the female close to the ground at the bottom of the nest box, where they allowed her to escape.

At the second capture (day 12 of incubation, “late incubation”), females were alternately assigned to either a plumage dulling or control signal manipulation treatment within each predation and age class. Note that the birds had only experienced either a predator treatment or a control treatment prior to their second capture, so all female fecal samples from Unit 1 and Unit 2 at the second capture were classified as either “Predator” or “Control.” For the plumage dulling treatment, researchers used a light grey non-toxic marker (Faber-Castell PITT artist pen ‘big brush’ warm grey III 272) to color the female’s ventral surface. This treatment dulled female ventral color within the natural range of variation in the population (Taff et al., 2021). For the control treatment, researchers colored females for the same amount of time and over the same area with a clear marker (Prismacolor premier colorless blender PB-121). These treatments were reapplied on the third capture during provisioning. For more details about these treatments, see Taff et al. (2021). Fecal samples taken during the third (provisioning) capture were classified as either “Control,” “Predator/Control,” “Control/Dulling,” or “Predator/Dulling.”

The ALAN experiment included individuals from Turkey Hill and Unit 4. In this experiment, each box was fitted with an LED light on the underside of the box top. For experimental nests, this light turned on at night starting one day before incubation began and remained on until all nestlings fledged. For control nests, the light stayed off. Females in this experiment were captured once during incubation (day 6-7) and once during provisioning (day 6-7). Females in this experiment were also captured once at night, just after their eggs hatched, for melatonin sampling, but there were not enough fecal samples collected during this capture, so they are excluded from this paper. For more details of this experiment, see Injaian et al. (2021). Note that, because this treatment started before the first incubation capture, all fecal samples taken at Unit 4 and Turkey Hill were classified as either “Light” or “Control” for both the incubation capture and the provisioning capture.

*Relationship between proportion aquatic arthropods via relative read abundance versus occurrence*: After calculating proportion aquatic with both relative abundance and occurrence, we examined the relationship between these two metrics. A linear model showed that proportion aquatic calculated via relative abundance predicts proportion aquatic calculated via occurrence (*β* = 0.24, SE = 0.02, p < 0.001, df = 427); however, the R^2^ values are relatively low (multiple R^2^ = 0.25, adjusted R^2^ = 0.25).

When proportion aquatic calculated via relative abundance is close to 100%, proportion aquatic calculated via occurrence is on average below 75% (Fig. S3), suggesting that, when terrestrial arthropods are uncommon by volume in the diet, proportion aquatic calculated via occurrence may overestimate how much of the diet is actually composed of them. Deagle et al. (2019) note that solely focusing on the occurrence of taxa, rather than their relative abundance, can overestimate the importance of food items consumed in lower amounts, including possible contaminants. Indeed, even after our filtering steps, the reads for some samples included mites and springtails, some of which likely inhabited the tree swallows’ nesting material. These arthropods were probably not consumed intentionally, nor were they consumed in large quantities. Additionally, Mengelkoch et al. (2004) found that tree swallow nestling diets were mostly aquatic arthropods in terms of biomass, however, the actual number of aquatic versus terrestrial arthropods was similar, suggesting that using proportion aquatic calculated via occurrence data would overestimate the amount of terrestrial arthropod biomass actually consumed by the birds.

**REFERENCES**:

Deagle, B. E., Thomas, A. C., McInnes, J. C., Clarke, L. J., Vesterinen, E. J., Clare, E. L., Kartzinel, T. R., & Eveson, J. P. (2019). Counting with DNA in metabarcoding studies: How should we convert sequence reads to dietary data? *Molecular Ecology*, *28*(2), 391–406. https://doi.org/10.1111/mec.14734

Injaian, A. S., **Uehling, J. J.,** Taff, C. C., & Vitousek, M. N. (2021) Effects of artificial light at night on avian provisioning, corticosterone, and reproductive success. *Integrative and Comparative Biology*, *61*(3):1147-1159. https://doi.org/10.1093/icb/icab055

Mengelkoch, J. M., Niemi, G. J., & Regal, R. R. (2004). Diet of the nestling tree swallow. *The Condor*, *106*(2), 423–429. https://doi.org/10.1093/condor/106.2.423

Taff, C. C., Zimmer, C., Scheck, D., Ryan, T. A., Houtz, J. L., Smee, M. R., Hendry, T. A., & Vitousek, M. N. (2021) Plumage manipulation alters associations between behaviour, physiology, the internal microbiome and fitness. *Animal Behavior*, *178*, 11-36. https://doi.org/10.1016/j.anbehav.2021.05.012

**FIGURES**:

**Figure S1**: A. Map of the four sites sampled. The large map shows the four sampling sites, with the scale bar shown at the bottom left. The inset map shows a zoomed-out view of the location of the sampling sites within New York state. The small pink box in the inset map shows the specific geographic region shown in the larger map. B. Zoomed in views of each specific site sampled. For Figs. S1A and S1B, background satellite imagery is from Google Maps. The outline of US states in Fig. S1A is from the US Census Bureau.

A

B

**Figure S2**: The distribution of samples across different numbers of read. The x-axis shows the log number of reads per sample, and the y-axis shows the number of samples that had that number of reads. Adult samples, negative controls, and nestling samples are separated. The red dotted line indicates 100 reads.


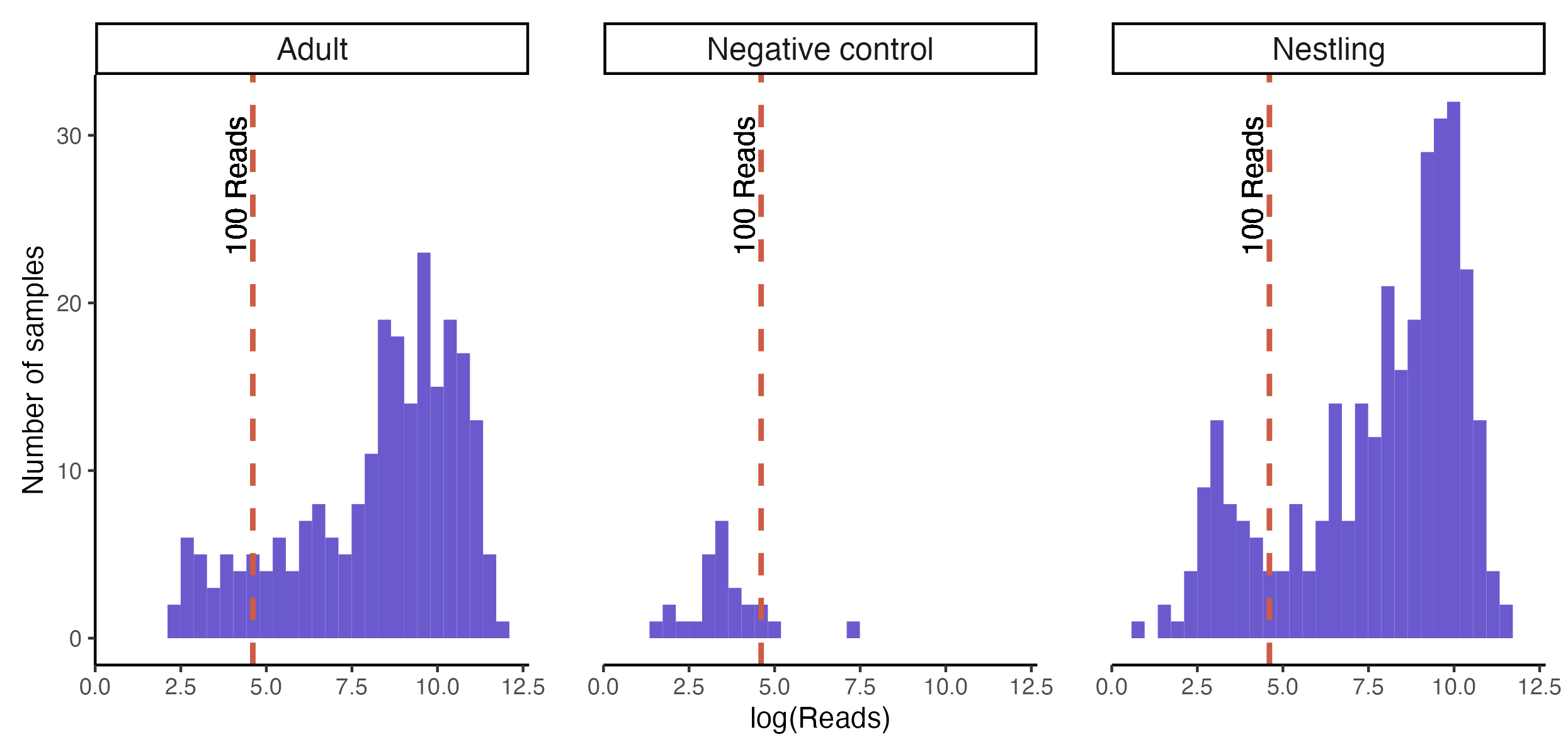


**Figure S3**: Relationship between the proportion of the diet composed of aquatic arthropods calculated via occurrence and via relative read abundance, including samples from both adults and nestlings. The blue line indicates the predicted relationship with a linear model, and the gray shaded area indicates the 95% confidence interval. The dashed black line shows a 1:1 relationship. Black dots are raw data from individual samples.

**
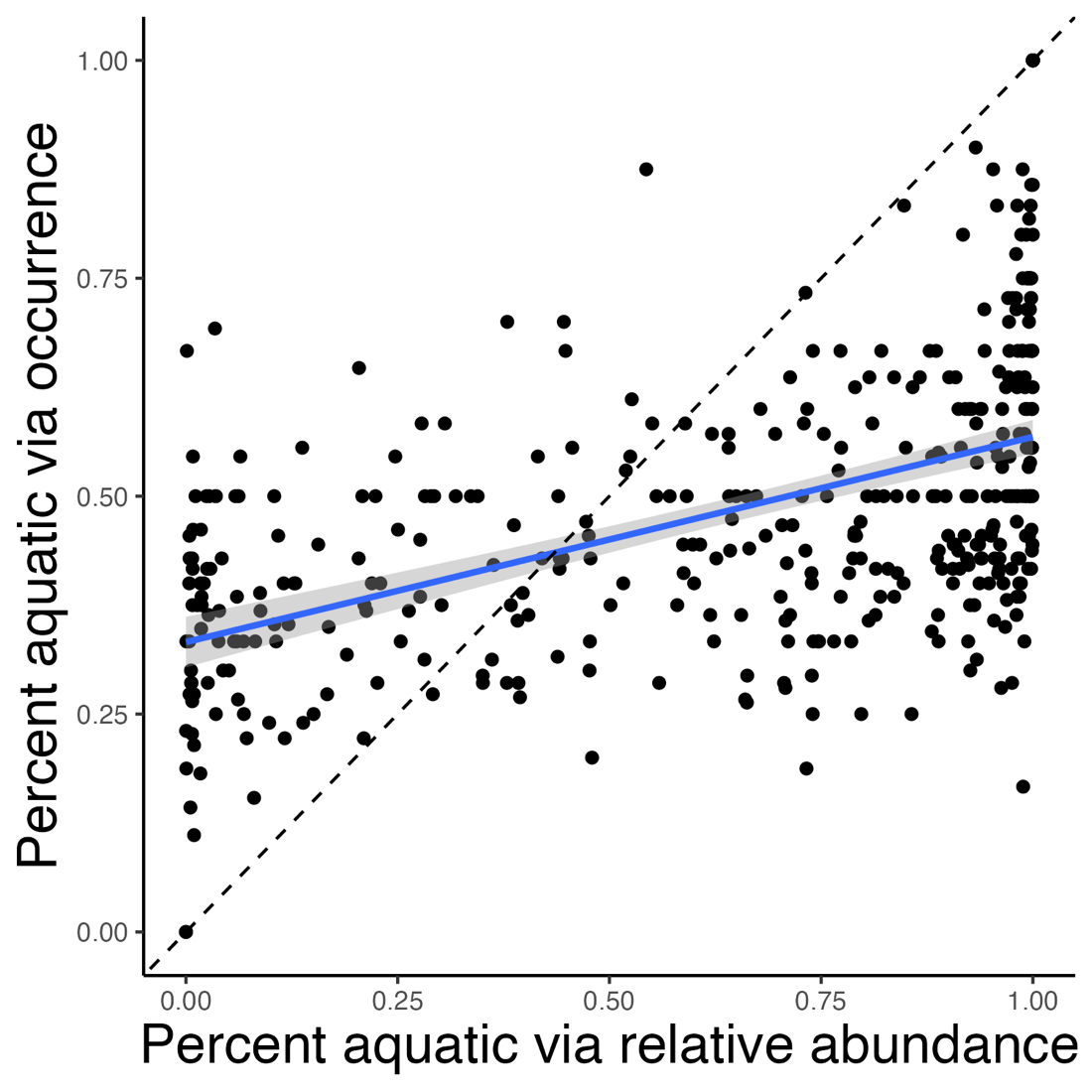
**

**TABLES**:

**Table S1**: After filtering steps, the number of fecal samples from adult females and adult males across breeding stages and sites included in the dataset.

|  | Unit 1 | Unit 2 | Unit 4 | Turkey Hill | Total |
| --- | --- | --- | --- | --- | --- |
| Adult females | | | | | |
| Early incubation | 25 | 32 | 8 | 9 | 74 |
| Late incubation | 3 | 20 | 0 | 0 | 23 |
| Provisioning | 6 | 30 | 6 | 3 | 45 |
|  | | | | **Total:** | **142** |
| Adult males | | | | | |
| Provisioning | 21 | 25 | 0 | 0 | 46 |
|  | | | | **Total:** | **46** |

*Note: one male was captured twice accidentally, with the first capture occurring during incubation. Here, only that male’s provisioning capture is tallied.

**Table S2**: After filtering steps, the number of fecal samples from nestlings across ages and sites in the dataset. The number of boxes from which fecal samples were collected is listed in parentheses after the site.

|  | Unit 1 (9 boxes) | Unit 2 (32 boxes) | Unit 4 (13 boxes) | Turkey Hill (7 boxes) | Total |
| --- | --- | --- | --- | --- | --- |
| Day 6 nestlings | 0 | 0 | 30 | 16 | 46 |
| Day 12 nestlings | 19 | 59 | 40 | 23 | 141 |
| Day 15 nestlings | 9 | 44 | 0 | 0 | 53 |
|  | | | | **Total:** | **240** |

**Table S3**: Results of an LMM examining the relationship between adult phenotype, site, brood size, nestling age, and Simpson’s diversity index of nestling diets. Bolded p-values indicate values below 0.05, which we consider statistically significant.

|  | **Simpson’s diversity index** | | | |
| --- | --- | --- | --- | --- |
| *Predictors* | *Estimates* | *95% confidence interval* | *p* | *df* |
| (Intercept) [Turkey Hill, Nestling day 6] | 0.37 | 0.23 – 0.50 | **<0.001** | 18.10 |
| Site [Unit 1] | 0.03 | -0.14 – 0.20 | 0.723 | 41.35 |
| Site [Unit 2] | -0.01 | -0.14 – 0.12 | 0.863 | 52.39 |
| Site [Unit 4] | 0.14 | 0.01 – 0.26 | **0.031** | 21.17 |
| Adult mass | 0.02 | -0.04 – 0.07 | 0.543 | 39.26 |
| Adult flat wing | -0.01 | -0.05 – 0.03 | 0.460 | 27.91 |
| Brood size at sampling time | 0.01 | -0.03 – 0.05 | 0.717 | 44.47 |
| Nestling day 12 | 0.09 | -0.02 – 0.19 | 0.097 | 198.34 |
| Nestling day 15 | 0.07 | -0.07 – 0.21 | 0.315 | 198.46 |

**Table S4**: Results of an LMM examining the relationship between adult phenotype, site, brood size, nestling age, and the proportion of the nestling diet composed of aquatic arthropods, calculated via relative abundance. Bolded p-values indicate values below 0.05, which we consider statistically significant.

|  | **Proportion aquatic (relative abundance, logit transformed)** | | | |
| --- | --- | --- | --- | --- |
| *Predictors* | *Estimates* | *95% confidence interval* | *p* | *df* |
| (Intercept) [Turkey Hill, Nestling day 6] | 2.94 | 0.92 – 4.96 | **0.007** | 18.88 |
| Site [Unit 1] | 0.23 | -2.50 – 2.96 | 0.866 | 44.65 |
| Site [Unit 2] | 0.77 | -1.31 – 2.85 | 0.459 | 43.13 |
| Site [Unit 4] | -0.74 | -2.76 – 1.27 | 0.458 | 32.17 |
| Adult mass | -0.16 | -0.93 – 0.62 | 0.690 | 46.43 |
| Adult flat wing | -0.21 | -0.87 – 0.45 | 0.520 | 37.76 |
| Brood size at sampling time | 0.11 | -0.50 – 0.72 | 0.719 | 62.82 |
| Nestling day 12 | -3.37 | -4.51 – -2.22 | **<0.001** | 183.16 |
| Nestling day 15 | -1.84 | -3.43 – -0.24 | **0.024** | 187.28 |

**Table S5**: Results of an LMM examining the relationship between adult phenotype, site, brood size, and nestling age and the proportion of the nestling diet composed of aquatic arthropods, calculated via occurrence. Bolded p-values indicate values below 0.05, which we consider statistically significant.

|  | **Proportion aquatic (occurrence, logit transformed)** | | | |
| --- | --- | --- | --- | --- |
| *Predictors* | *Estimates* | *95% confidence interval* | *p* | *df* |
| Intercept) [Turkey Hill, Nestling day 6] | 0.88 | 0.16 – 1.61 | **0.019** | 18.11 |
| Site [Unit 1] | -0.08 | -1.02 – 0.87 | 0.870 | 41.39 |
| Site [Unit 2] | 0.37 | -0.36 – 1.10 | 0.313 | 52.27 |
| Site [Unit 4] | -0.44 | -1.11 – 0.24 | 0.193 | 21.29 |
| Adult mass | 0.08 | -0.20 – 0.37 | 0.552 | 39.37 |
| Adult flat wing | -0.07 | -0.29 – 0.15 | 0.542 | 28.05 |
| Brood size at sampling time | 0.12 | -0.11 – 0.34 | 0.298 | 44.67 |
| Nestling day 12 | -1.34 | -1.90 – -0.78 | **<0.001** | 198.26 |
| Nestling day 15 | -0.91 | -1.68 – -0.14 | **0.021** | 198.40 |

**Table S6**: Results of an LMM examining the relationship between adult phenotype (flat wing and mass), site, breeding stage, the interaction between mass and breeding stage, and Simpson’s diversity index of adult diets. Bolded p-values indicate values below 0.05, which we consider statistically significant.

|  | **Simpson’s diversity index** | | | |
| --- | --- | --- | --- | --- |
| *Predictors* | *Estimates* | *95% confidence interval* | *p* | *df* |
| (Intercept) [Turkey Hill, mid incubation stage] | 0.31 | 0.10 – 0.51 | **0.005** | 17.49 |
| Site [Unit 1] | 0.11 | -0.07 – 0.28 | 0.227 | 75.63 |
| Site [Unit 2] | 0.11 | -0.05 – 0.28 | 0.183 | 78.16 |
| Site [Unit 4] | 0.15 | -0.04 – 0.34 | 0.119 | 97.68 |
| Flat wing | -0.00 | -0.05 – 0.04 | 0.853 | 75.79 |
| Mass | 0.01 | -0.08 – 0.10 | 0.801 | 130.18 |
| Late incubation stage | -0.02 | -0.20 – 0.15 | 0.808 | 26.91 |
| Provisioning stage | -0.14 | -0.33 – 0.05 | 0.148 | 79.48 |
| Mass * late incubation stage | -0.14 | -0.34 – 0.06 | 0.181 | 109.32 |
| Mass * provisioning stage | -0.22 | -0.37 – -0.08 | **0.003** | 114.81 |

**Table S7**: Results of an LMM examining the relationship between adult phenotype (including mass change, rather than mass), site, and Simpson’s diversity index of adult diets. Bolded p-values indicate values below 0.05, which we consider statistically significant.

|  | **Simpson’s diversity index** | | | |
| --- | --- | --- | --- | --- |
| *Predictors* | *Estimates* | *95% confidence interval* | *p* | *df* |
| (Intercept) [Turkey Hill] | 0.27 | -0.05 – 0.59 | 0.099 | 34.42 |
| Site [Unit 1] | 0.17 | -0.20 – 0.54 | 0.352 | 38.62 |
| Site [Unit 2] | 0.24 | -0.09 – 0.58 | 0.152 | 38.39 |
| Site [Unit 4] | 0.11 | -0.25 – 0.46 | 0.543 | 35.93 |
| Flat wing | -0.06 | -0.13 – 0.02 | 0.156 | 37.10 |
| Mass change | -0.05 | -0.13 – 0.04 | 0.260 | 37.19 |

**Table S8**: Results of an LMM examining the relationship between adult phenotype (flat wing and mass), site, breeding stage, the interaction between mass and breeding stage, and the proportion of the adult diet composed of aquatic arthropods, calculated via relative abundance. Bolded p-values indicate values below 0.05, which we consider statistically significant.

|  | **Proportion aquatic (relative abundance, logit transformed)** | | | |
| --- | --- | --- | --- | --- |
| *Predictors* | *Estimates* | *95% confidence interval* | *p* | *df* |
| (Intercept) [Turkey Hill, mid incubation stage, Mass * mid incubation stage] | 4.61 | 2.13 – 7.10 | **0.001** | 15.25 |
| Site [Unit 1] | -1.48 | -3.57 – 0.62 | 0.165 | 76.74 |
| Site [Unit 2] | -1.45 | -3.40 – 0.50 | 0.142 | 80.19 |
| Site [Unit 4] | -4.80 | -7.08 – -2.51 | **<0.001** | 100.38 |
| Adult flat wing | 0.27 | -0.26 – 0.81 | 0.313 | 70.00 |
| Adult mass | -0.06 | -1.13 – 1.01 | 0.914 | 129.44 |
| Late incubation stage | 0.13 | -2.05 – 2.30 | 0.905 | 25.89 |
| Provisioning stage | -1.16 | -3.52 – 1.20 | 0.331 | 73.67 |
| Mass * Late incubation stage | 0.64 | -1.88 – 3.15 | 0.618 | 123.57 |
| Mass * Provisioning stage | -0.16 | -1.99 – 1.67 | 0.860 | 122.01 |

**Table S9**: Results of an LMM examining the relationship between adult phenotype (flat wing and mass), site, breeding stage, the interaction between mass and breeding stage, and the proportion of the adult diet composed of aquatic arthropods, calculated via occurrence. Bolded p-values indicate values below 0.05, which we consider statistically significant.

|  | **Proportion aquatic (occurrence, logit transformed)** | | | |
| --- | --- | --- | --- | --- |
| *Predictors* | *Estimates* | *95% confidence interval* | *p* | *df* |
| (Intercept) [Turkey Hill, Mid incubation stage, Mass * Mid incubation stage] | 0.86 | -0.82 – 2.55 | 0.292 | 15.25 |
| Site [Unit 1] | 0.02 | -1.40 – 1.44 | 0.981 | 76.74 |
| Site [Unit 2] | -0.19 | -1.51 – 1.13 | 0.770 | 80.19 |
| Site [Unit 4] | -1.00 | -2.54 – 0.55 | 0.204 | 100.38 |
| Adult flat wing | 0.27 | -0.10 – 0.63 | 0.148 | 70.00 |
| Adult mass | 0.03 | -0.69 – 0.75 | 0.931 | 129.44 |
| Late incubation stage | 0.22 | -1.26 – 1.69 | 0.767 | 25.89 |
| Provisioning stage | -0.24 | -1.84 – 1.36 | 0.762 | 73.67 |
| Mass * Late incubation stage | 0.06 | -1.65 – 1.76 | 0.947 | 123.57 |
| Mass * Provisioning stage | 0.15 | -1.08 – 1.39 | 0.806 | 122.01 |

**Table S10**: Results of an LMM examining the relationship between adult phenotype (including mass change, rather than mass), site, and the proportion of the adult diet composed of aquatic arthropods, calculated via relative abundance. Bolded p-values indicate values below 0.05, which we consider statistically significant.

|  | **Proportion aquatic (relative abundance, logit transformed)** | | | |
| --- | --- | --- | --- | --- |
| *Predictors* | *Estimates* | *95% confidence interval* | *p* | *df* |
| (Intercept) [Turkey Hill] | 5.62 | 2.64 – 8.59 | **0.001** | 34.60 |
| Site [Unit 1] | -3.45 | -6.93 – 0.03 | 0.052 | 38.72 |
| Site [Unit 2] | -3.44 | -6.56 – -0.32 | **0.032** | 36.69 |
| Site [Unit 4] | -7.49 | -10.86 – -4.11 | **<0.001** | 36.21 |
| Adult flat wing | -0.20 | -0.97 – 0.56 | 0.591 | 38.83 |
| Adult mass change | -0.21 | -1.00 – 0.58 | 0.593 | 38.81 |

**Table S11**: Results of an LMM examining the relationship between adult phenotype (including mass change, rather than mass), site, and the proportion of the adult diet composed of aquatic arthropods, calculated via occurrence. Bolded p-values indicate values below 0.05, which we consider statistically significant.

|  | **Proportion aquatic (occurrence, logit transformed)** | | | |
| --- | --- | --- | --- | --- |
| *Predictors* | *Estimates* | *95% confidence interval* | *p* | *df* |
| (Intercept) [Turkey Hill] | 2.84 | 1.15 – 4.54 | **0.002** | 34.60 |
| Site [Unit 1] | -2.95 | -4.93 – -0.97 | **0.005** | 38.72 |
| Site [Unit 2] | -2.79 | -4.56 – -1.01 | **0.003** | 36.69 |
| Site [Unit 4] | -3.09 | -5.01 – -1.17 | **0.002** | 36.21 |
| Adult flat wing | 0.24 | -0.19 – 0.68 | 0.262 | 38.83 |
| Adult mass change | 0.02 | -0.43 – 0.47 | 0.916 | 38.81 |

**Table S12:** Results of LMMs examining the relationships between age and sex (adult female, adult male, and nestling) and diet characteristics. Results from three models are shown. Bolded p-values indicate values below 0.05, which we consider statistically significant.

|  | **Simpson’s diversity index** | | | |
| --- | --- | --- | --- | --- |
| *Predictors* | *Estimates* | *95% confidence interval* | *p* | *df* |
| (Intercept) [Adult female] | 0.48 | 0.41 – 0.56 | **<0.001** | 92.81 |
| Bird category [Adult male] | -0.02 | -0.12 – 0.09 | 0.733 | 300.26 |
| Bird category [Nestling] | -0.02 | -0.10 – 0.07 | 0.667 | 299.61 |
|  | **Proportion aquatic (relative abundance, logit transformed)** | | | |
| *Predictors* | *Estimates* | *95% confidence interval* | *p* | *df* |
| (Intercept) [Adult female] | 1.84 | 0.82 – 2.85 | **0.001** | 44.63 |
| Bird category [Adult male] | -1.07 | -2.37 – 0.23 | 0.107 | 292.50 |
| Bird category [Nestling] | -1.07 | -2.09 – -0.04 | **0.042** | 298.57 |
|  | **Proportion aquatic (occurrence family, logit transformed)** | | | |
| *Predictors* | *Estimates* | *95% confidence interval* | *p* | *df* |
| (Intercept) [Adult female] | 0.17 | -0.29 – 0.63 | 0.459 | 70.82 |
| Bird category [Adult male] | -0.50 | -1.12 – 0.11 | 0.109 | 302.75 |
| Bird category [Nestling] | -0.30 | -0.78 – 0.18 | 0.224 | 308.14 |
